# Structure-based design, synthesis and crystallization of 2-arylquinazolines as lipid pocket ligands of p38α MAPK

**DOI:** 10.1371/journal.pone.0184627

**Published:** 2017-09-11

**Authors:** Mike Bührmann, Bianca M. Wiedemann, Matthias P. Müller, Julia Hardick, Maria Ecke, Daniel Rauh

**Affiliations:** Faculty of Chemistry and Chemical Biology, TU Dortmund University, Dortmund, Germany; University of Oslo, NORWAY

## Abstract

In protein kinase research, identifying and addressing small molecule binding sites other than the highly conserved ATP-pocket are of intense interest because this line of investigation extends our understanding of kinase function beyond the catalytic phosphotransfer. Such alternative binding sites may be involved in altering the activation state through subtle conformational changes, control cellular enzyme localization, or in mediating and disrupting protein-protein interactions. Small organic molecules that target these less conserved regions might serve as tools for chemical biology research and to probe alternative strategies in targeting protein kinases in disease settings. Here, we present the structure-based design and synthesis of a focused library of 2-arylquinazoline derivatives to target the lipophilic C-terminal binding pocket in p38*α* MAPK, for which a clear biological function has yet to be identified. The interactions of the ligands with p38*α* MAPK was analyzed by SPR measurements and validated by protein X-ray crystallography.

## Introduction

Protein kinases have been a frequent topic in medicinal chemistry and drug development due to their key function as mediating components in signal transduction, regulating cellular pathways on a molecular level, thereby playing a crucial role in the emergence of several diseases. The conventional approach towards the treatment of kinase-related diseases has involved the administration of ATP-competitive inhibitors which potently occupy and thereby block the enzyme’s active site where the phosphotransfer from ATP to target substrates takes place [1, 2]. However, development of specifically selective inhibitors for a certain targeted kinase within the related members of this enzyme family remains a major hurdle in drug research [3, 4].

Successful strategies to gain improved selectivity within the kinome have revolved around employing unique structural features of individual kinases, such as covalent modification of cysteines [5, 6] or identifying and targeting alternative binding pockets distant from the active site [7]. Alternative bindings sites far from the ATP-pocket can directly regulate kinase affinity and can potentially be addressed by small molecules which alter the kinase activity in a dual manner, *via* both inhibition and activation [8, 9].

In addition to advantages in the development of selective kinase modulators, these binding sites can aid in distinguishing so called non-catalytic functions, those processes triggered by protein-protein (or protein-target) interactions, where kinases serve as scaffolds, *e*.*g*., for the formation of multi-enzyme-complexes [10]. In this way, these remote sites can serve as (allosteric) effectors of target molecules, directly affecting the location or the activity state of interaction partners, or can influence cell proliferation, differentiation, and apoptosis. An increasing number of those scaffolding functions of kinases are gradually being discovered and their functions may even exceed the significance of the solely catalytic properties [10–12].

**Fig 1:**
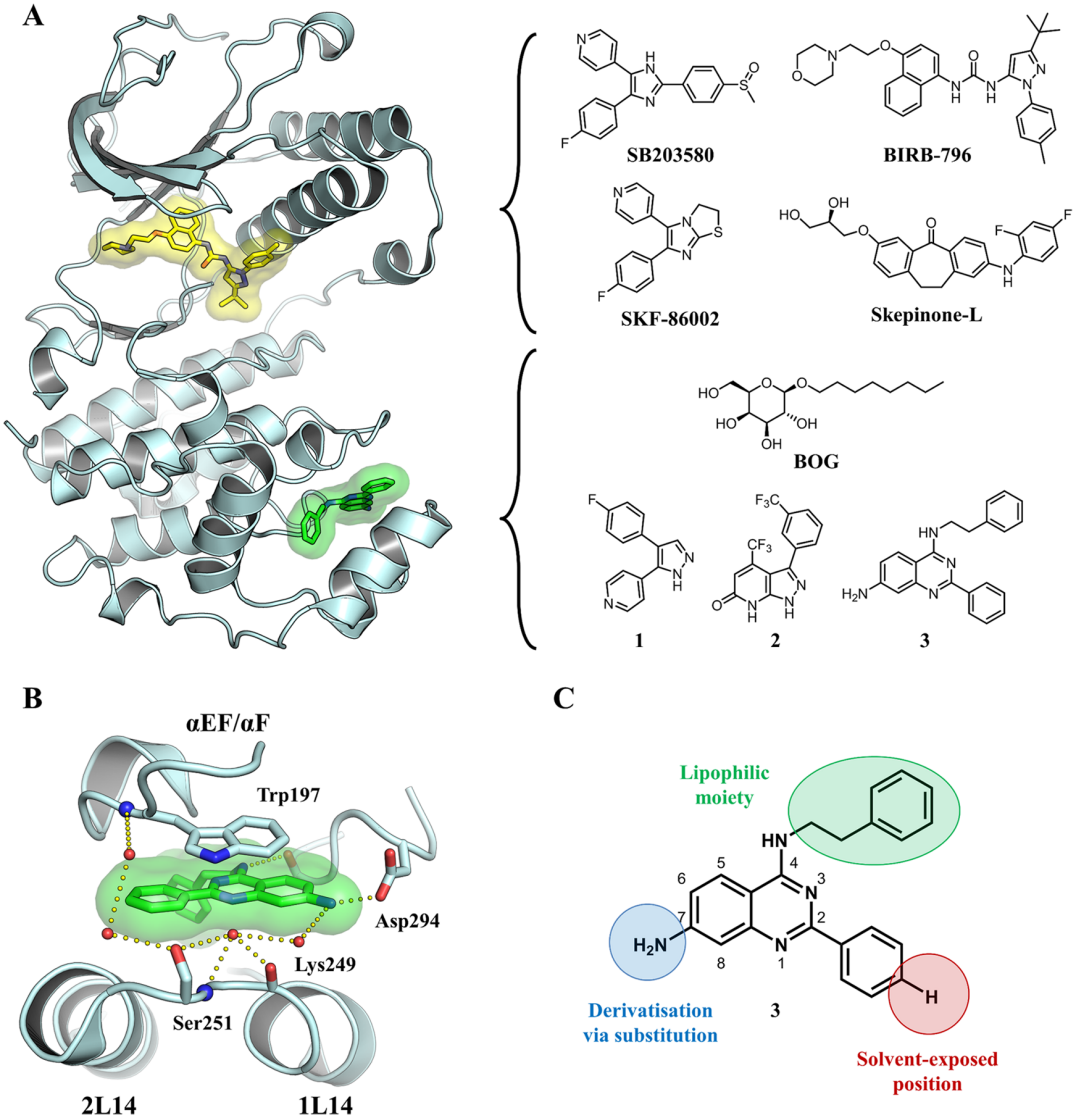
Binding modes of active site inhibitors and LiPoLis in p38*α* MAPK. (A) Superposed kinase domains of p38*α* MAPK (cyan) in complex with active site inhibitor BIRB-796 (yellow) (PDB: 1KV2) and the quinazoline-based LiPoLi **3** (green) (PDB: 4DLJ). (B) Detailed binding mode of **3** (green) in the LP of p38*α* MAPK (cyan), highlighting key structural elements and main interactions formed between the protein and the ligand. (C) Chemical structure of **3** with systematic numbering of the quinazoline scaffold and highlighted moieties selected for derivatization.

Accordingly, the identification and exploration of non-conserved regions may provide insights into the putative unknown functions of protein kinases beyond catalysis and allosteric regulation. Thus, elucidation of unique structural features that modulate protein kinases through employing alternative binding pockets and investigations of the design of corresponding small molecules as alternatives to ATP-competitive ligands has moved to the forefront of kinase inhibitor research and kinase biology. Against this background, we recently identified a novel class of p38*α* MAPK (mitogen-activated protein kinase) binders addressing a C-terminal lipophilic binding pocket (LP) accessible for small molecules located at several angstroms distance from the enzymes active site. The discovered 2-arylquinazolines bind to the LP and their corresponding co-crystal structures revealed a very distinct binding mode of these lipid pocket ligands (LiPoLis) to p38*α* (Fig 1B). We considered that these ligands may serve as interesting starting points to study the yet unexplored functions of this binding site in p38*α* [13]. Several small molecules have been described to address this pocket (Fig 1A) and they can be classified into detergent-like molecules, *β*-octyl-D-glucopyranoside (BOG) [14] and phosphatidylinositol ether lipid analogues [15], and small organic compounds, **1** (4-[3-(4-fluorophenyl)-1*H*-pyrazol-4-yl]pyridine) [16] and **2** (4-(trifluoromethyl)-3-(3-(trifluoromethyl)phenyl)-1*H*-pyrazolo[3,4-*b*]pyridine-6(7*H*)-one) (Fig 1A) [17]. Overall, the majority of the published compounds that address the LP have exhibited a rather low affinity towards p38*α* [17, 18].

The LP consists of the two *α*-helices 1L14 and 2L14, the *α*EF/*α*F loop and a deep lipophilic sub-pocket that is mostly decorated with hydrophobic amino acid side chains (Fig 1B). Based on published co-crystal structures and biochemical assay data, there are indications that some of the already described LiPoLis alter the kinase conformation in a way that the enzymatic activity could be directly influenced by ligand binding, although the corresponding data was measured at high concentrations [15–17]. Previous publications also speculated that there might be a regulatory fine-tuning as a result of conformational changes within the LP when addressed by ligands [15, 18]. Thus, the biological role of the LP is still not fully understood and may well serve some yet unknown function [19].

To gain a more detailed insight into the role of the p38*α* LP, we undertook SAR studies based on our previously found lead structure **3** and the known binding mode. Here, we present a structure-based design, synthesis, and validation by surface plasmon resonance (SPR) analysis and protein X-ray crystallography of novel LiPoLis that target the LP in p38*α* MAPK.

## Materials and methods

### Reagents and materials

Unless otherwise noted, all reagents and solvents were purchased from Acros, Alfa Aesar, Apollo Scientific, Fluka, Merck or Sigma-Aldrich and used without further purification. Dry solvents were purchased as anhydrous reagents from commercial suppliers. ^1^H and ^13^C spectra were recorded on a Bruker Avance DRX 400 and a Bruker Avance DRX 500 spectrometer at 400 MHz or 500 MHz and 101 MHz or 125 MHz, respectively. Chemical shifts are reported in *δ* (ppm) as s (singlet), d (doublet), dd (doublet of doublet), t (triplet), q (quartet), m (multiplet) and bs (broad singlet) and are referenced to the residual solvent signal: DMSO-*d*_*6*_ (2.50) or CDCl_3_ (7.26) for ^1^H and DMSO-*d*_*6*_ (39.52) or CDCl_3_ (77.16) for ^13^C. Compound identity was further confirmed by LC-MS analysis on LCQ Advantage MAX (1200 series, Agilent) with Eclipse XDB-C18-column (5 μM, 150 × 1.6 mm, Phenomenex). High resolution electrospray ionization mass spectra (ESI-FTMS) were recorded on a Thermo LTQ Orbitrap (high resolution mass spectrometer from Thermo Electron) coupled to an "Accela" HPLC system supplied with a "Hypersil GOLD" column (Thermo Electron). Analytical TLC was carried out on Merck 60 F245 aluminium-backed silica gel plates. Compounds were purified by column chromatography using silica gel from Baker (40–70 μm particle size) or VWR Prolabo (Normasil 60, 40–63 μm particle size). Flash column chromatography was done using a Biotage Isolera One system with Biotage SNAP and SNAP Ultra columns, respectively, and monitored by UV at 254 and 360 nm. Preparative HPLC was conducted on a Varian HPLC system (Pro Star 215) with a VP 250/21 Nucleosil C18 PPN column from Macherey-Nagel and monitored by UV at 254 nm. All presented compounds were analyzed by HPLC to determine and ensure a purity of ≥ 95%.

### Synthesis and analytics

#### Preparation of 7-nitro-2-phenylquinazolin-4-ol (7a)

To a solution of **5a** (5 g, 27.5 mmol) and benzamidine hydrochloride (3 g, 24.9 mmol) in 2-methoxyethanol (150 mL) was added AcOH (3 mL) and the reaction mixture was stirred at 130°C for 18 h. The warm solution was subsequently filtered and the residue washed with cold MeOH to obtain **7a** as a pale brown solid (1.8 g, 24%). ^**1**^**H NMR** (400 MHz, DMSO-*d*_*6*_) *δ* 12.93 (s, 1H), 8.43 (d, *J* = 2.1 Hz, 1H), 8.36 (d, 1H), 8.24 (d, *J* = 2.2 Hz, 1H), 8.24–8.20 (m, 2H), 7.65 (t, *J* = 7.3 Hz, 1H), 7.59 (t, *J* = 7.4 Hz, 2H); ^**13**^**C NMR** (126 MHz, DMSO-*d*_*6*_) *δ* 161.3, 154.5, 151.3, 149.1, 132.1, 131.9, 128.6, 128.1, 128.0, 125.3, 122.4, 112.0; **HRMS** (ESI-MS): Calculated for C_14_H_10_O_3_N_3_ [M+H]^+^: 268.07167; found: 268.07159.

#### Preparation of 8-nitro-2-phenylquinazolin-4-ol (7b)

Anthranilic acid **5b** (500 mg, 2.8 mmol) was placed in a microwave reaction vial and benzoic anhydride was added (1.55 g, 6.9 mmol). Prior to adding formamide (1.1 mL, 27.5 mmol) benzoic acid was melted at 45°C to homogenize the reaction mixture. After 10 min heating at 200°C in a microwave reactor, the solution was poured into ice-water and stirred for 10 min. Subsequent filtration yielded the product **7b** (268 mg, 37%). ^**1**^**H NMR** (500 MHz, DMSO-*d*_*6*_) *δ* 12.93 (s, 1H), 8.38 (dd, *J* = 8.0, 1.4 Hz, 1H), 8.31 (dd, *J* = 7.8, 1.4 Hz, 1H), 8.18–8.13 (m, 2H), 7.67–7.61 (m, 2H), 7.60–7.54 (m, 2H); ^**13**^**C NMR** (101 MHz, DMSO-*d*_*6*_) *δ* 160.97, 154.65, 146.68, 140.55, 132.23, 131.95, 129.72, 128.77, 128.24, 128.16, 126.02, 122.54.

### Common procedure for the generation of 2-arylquinazoline scaffolds (7c-e)

Anthranilic amide **6** (1 eq), NaHSO_3_ (1.2 eq) and a corresponding aldehyde building block (1 eq) were dissolved in DMAc and the solution stirred for 30 min. *p*TSA (0.1 eq) was added and the mixture was stirred at 155°C for 18 h. After concentration of the reaction mixture *in vacuo* and separation between saturated NaHCO_3_ and DCM, the crude product was purified by flash column chromatography to isolate the desired 2-arylquinazoline.

#### Preparation of 1-(4-(4-(4-hydroxy-7-nitroquinazolin-2-yl)phenyl)piperazin-1-yl)ethan-1-one (7c)

According to the common procedure, amide **6** (1 g, 5.5 mmol), NaHSO_3_ (690 mg, 6.6 mmol), aldehyde (1.3 g, 5.6 mmol) and *p*TSA (100 mg, 0.5 mmol) in DMAc (50 mL) were converted to a crude reaction mixture that was purified by flash column chromatography (0% → 2% MeOH/DCM) to give 390 mg (18%) of the described target compound. ^**1**^**H NMR** (500 MHz, DMSO-*d*_*6*_) *δ* 12.58 (s, 1H), 8.34 (d, *J* = 2.1 Hz, 1H), 8.30 (d, *J* = 8.7 Hz, 1H), 8.16 (d, *J* = 9.0 Hz, 2H), 8.12 (dd, *J* = 8.7, 2.2 Hz, 1H), 7.06 (d, *J* = 9.0 Hz, 2H), 3.67–3.54 (m, 4H), 3.43–3.38 (m, 2H), 3.34–3.29 (m, 2H), 2.05 (s, 3H); ^**13**^**C NMR** (126 MHz, DMSO-*d*_*6*_) *δ* 168.4, 154.1, 152.8, 151.3, 129.3, 128.1, 120.5, 119.0, 113.7, 46.8, 46.5, 45.0, 40.4, 21.2; **HRMS** (ESI-MS): Calculated for C_20_H_20_O_4_N_5_ [M+H]^+^: 393.15098; found: 393.15035.

#### Preparation of 2-(4-morpholinophenyl)-7-nitroquinazolin-4-ol (7d)

Following the common procedure, amide **6** (2.2 g, 12.1 mmol), NaHSO_3_ (1.5 g, 14.4 mmol), aldehyde (2.3 g, 12.1 mmol) and *p*TSA (230 mg, 1.2 mmol) in DMAc (50 mL) were converted to a crude reaction mixture that was purified by flash column chromatography (0% → 2% MeOH/DCM) to give 430 mg (10%) of the described target compound. ^**1**^**H NMR** (600 MHz, DMSO-*d*_*6*_) δ 12.60 (s, 1H), 8.33 (s, 1H), 8.29 (dd, *J* = 8.7, 1.5 Hz, 1H), 8.17–8.09 (m, 3H), 7.05 (d, *J* = 7.5 Hz, 2H), 3.73 (s, 4H), 3.28 (s, 4H); ^**13**^**C NMR** (101 MHz, DMSO-*d*_*6*_) *δ* 153.4, 151.4, 129.3, 128.2, 120.8, 119.1, 115.9, 115.7, 113.6, 65.9, 47.0; **HRMS** (ESI-MS): Calculated for C_18_H_17_O_4_N_4_ [M+H]^+^: 353.12443; found: 333.12444.

#### Preparation of 2-(4-(methylthio)phenyl)-7-nitroquinazolin-4-ol (7e)

According to the common procedure, amide **6** (400 mg, 2.2 mmol), NaHSO_3_ (270 mg, 2.6 mmol), aldehyde (420 mg, 2.2 mmol) and *p*TSA (40 mg, 0.2 mmol) in DMAc (5 mL) were converted and the crude product was subsequently used without further purification (235 mg, 34%). ^**1**^**H NMR** (400 MHz, DMSO-*d*_*6*_) *δ* 12.85 (s, 1H), 8.40 (d, *J* = 2.0 Hz, 1H), 8.34 (d, *J* = 8.7 Hz, 1H), 8.21 (d, *J* = 2.2 Hz, 1H), 8.19 (d, *J* = 2.8 Hz, 1H), 8.16 (s, 1H), 7.42 (d, *J* = 8.5 Hz, 2H), 2.56 (s, 3H); ^**13**^**C NMR** (101 MHz, DMSO-*d*_*6*_) *δ* 161.41, 153.99, 151.37, 149.28, 146.85, 144.03, 128.36, 128.19, 127.91, 125.25, 125.10, 122.30, 119.85, 14.04; **HRMS** (ESI-MS): Calculated for C_15_H_12_O_3_N_3_S [M+H]^+^: 314.05939; found: 314.05934.

### Common procedure for the preparation of 4-amino-7-/8-nitroquinazolines (8a-d)

Substituted quinazolines **8a-d** were synthesized following a common procedure. Quinazoline **7a** (1 eq) was dissolved in SOCl_2_ and catalytic amounts of DMF. The reaction mixture was stirred for 4 h at 80°C before the solvent was evaporated under reduced pressure. The residue was dissolved in DCM/*i*PrOH (3:2) before the corresponding amine (1.5 eq) and DIPEA (2 eq) were added. After stirring overnight at room temperature, extraction with saturated NaHCO_3_/EtOAc, concentration *in vacuo* and silica gel column chromatography yielded the described target compounds.

#### Preparation of *N*-benzyl-7-nitro-2-phenylquinazolin-4-amine (8a)

According to the general procedure, quinazoline **7a** (200 mg, 0.75 mmol) was converted to **8a** and the crude product was purified by recrystallization from MeOH to yield 211 mg (79%) of the described compound. ^**1**^**H NMR** (500 MHz, DMSO-*d*_*6*_) *δ* 9.32 (t, *J* = 5.7 Hz, 1H), 8.56 (d, *J* = 9.0 Hz, 1H), 8.48–8.42 (m, 3H), 8.22 (dd, *J* = 9.0, 2.4 Hz, 1H), 7.55–7.42 (m, 5H), 7.39–7.28 (m, 2H), 7.25 (t, *J* = 7.3 Hz, 1H), 4.94 (d, *J* = 5.7 Hz, 2H); ^**13**^**C NMR** (126 MHz, DMSO-*d*_*6*_) *δ* 161.2, 159.3, 150.1, 139.1, 137.7, 131.0, 128.3, 128.3, 128.1, 127.5, 125.2, 122.8, 118.5, 117.4, 44.1; **HRMS** (ESI-MS): Calculated for C_21_H_15_N_4_O_2_ [M+H]^+^: 357.13460; found: 357.13446.

#### Preparation of *N*-(4-fluorophenyl)-7-nitro-2-phenylquinazolin-4-amine (8b)

Following the general procedure, quinazoline **7a** (240 mg, 0.89 mmol) was converted to **8b** and the crude product was purified by recrystallization from MeOH to yield 213 mg (66%) of the described compound. ^**1**^**H NMR** (400 MHz, DMSO-*d*_*6*_) *δ* 10.22 (s, 1H), 8.71 (d, *J* = 9.0 Hz, 1H), 8.45 (d, *J* = 2.3 Hz, 1H), 8.42–8.36 (m, 2H), 8.24 (dd, *J* = 9.0, 2.3 Hz, 1H), 7.94–7.83 (m, 2H), 7.54–7.46 (m, 3H), 7.29 (t, *J* = 8.9 Hz, 2H); ^**13**^**C NMR** (101 MHz, DMSO-*d*_*6*_) *δ* 161.05, 158.42 (d, *J* = 240.7 Hz), 157.75, 150.64, 150.05, 137.82, 136.64, 130.71, 128.43, 128.11, 125.71, 124.65 (d, *J* = 8.0 Hz), 122.68, 118.72, 118.53, 115.06 (d, *J* = 22.2 Hz); **HRMS** (ESI-MS): Calculated for C_20_H_14_N_4_O_2_F [M+H]^+^: 361.10953; found: 361.10963.

#### Preparation of *N*-(4-fluorobenzyl)-7-nitro-2-phenylquinazolin-4-amine (8c)

Following the general procedure, quinazoline **7a** (200 mg, 0.75 mmol) was converted to **8c** and the crude product was purified by silica gel column chromatography (5 → 12% EtOAc/PE) to yield 265 mg (95%) of the described compound. ^**1**^**H NMR** (400 MHz, DMSO-*d*_*6*_) *δ* 9.33 (t, *J* = 5.7 Hz, 1H), 8.54 (d, *J* = 9.0 Hz, 1H), 8.46 (m, 3H), 8.22 (dd, *J* = 9.0, 2.3 Hz, 1H), 7.60–7.45 (m, 5H), 7.16 (t, *J* = 8.9 Hz, 2H), 4.91 (d, *J* = 5.6 Hz, 2H); ^**13**^**C NMR** (101 MHz, DMSO-*d*_*6*_) *δ* 161.28 (d, *J* = 242.4 Hz), 161.13, 159.28, 150.14, 137.68, 135.33, 130.79, 129.51 (d, *J* = 8.1 Hz), 128.40, 128.14, 125.24, 122.89, 118.65, 117.45, 115.14 (d, *J* = 21.3 Hz), 43.42; **HRMS** (ESI-MS): Calculated for C_21_H_16_N_4_O_2_F [M+H]^+^: 375.12518; found: 375.12529.

#### Preparation of *N*-(3-fluorophenyl)-7-nitro-2-phenylquinazolin-4-amine (8d)

Following the general procedure, quinazoline **7a** (240 mg, 0.89 mmol) was converted to **8d** and the crude product was purified by recrystallization from MeOH and precipitation in acetone to yield 233 mg (72%) of the described compound. ^**1**^**H NMR** (500 MHz, DMSO-*d*_*6*_) *δ* 10.27 (s, 1H), 8.78 (d, *J* = 9.1 Hz, 1H), 8.53 (d, *J* = 1.4 Hz, 1H), 8.45–8.38 (m, 2H), 8.34–8.24 (m, 1H), 7.96 (d, *J* = 11.8 Hz, 1H), 7.76 (d, *J* = 8.0 Hz, 1H), 7.58–7.23 (m, 4H), 7.10–7.02 (m, 1H); ^**13**^**C NMR** (126 MHz, DMSO-*d*_*6*_) *δ* 161.95 (d, *J* = 241.4 Hz), 160.85, 157.63, 150.54, 150.31, 140.51 (d, *J* = 11.1 Hz), 137.34, 131.00, 130.08 (d, *J* = 9.5 Hz), 128.55, 128.06, 125.58, 123.09, 119.10, 117.91, 117.55, 110.57 (d, *J* = 21.0 Hz), 109.02 (d, *J* = 26.2 Hz); **HRMS** (ESI-MS): Calculated for C_20_H_14_N_4_O_2_F [M+H]^+^: 361.10953; found: 361.11130.

### Common procedure for the preparation of 4-amino-7-/8-nitroquinazolines (8e-n)

Substituted quinazolines **8e-n** were synthesized following a common procedure. Quinazolines **7a-e** (1 eq) and HCCP (1 eq) were dissolved in DIPEA (5 eq) and MeCN. The reaction mixture was stirred for 60 min at rt before the corresponding amine (6 eq) was added. After further stirring for 18 h at rt, the crude products was purified by silica gel column chromatography to yield the described target compounds.

#### Preparation of *N*-(3,4-difluorophenyl)-7-nitro-2-phenylquinazolin-4-amine (8e)

According to the general procedure, quinazoline **7a** (150 mg, 0.56 mmol) was converted and the crude product purified by silica gel column chromatography (1% → 20% EtOAc/PE) to the substituted aminoquinazoline **8e** (114 mg, 56%). ^**1**^**H NMR** (500 MHz, DMSO-*d*_*6*_) *δ* 10.25 (s, 1H), 8.72 (d, *J* = 9.1 Hz, 1H), 8.49 (d, *J* = 2.1 Hz, 1H), 8.37 (m, 2H), 8.28 (dd, *J* = 9.0 Hz, 2.2 Hz,1H), 8.11 (ddd, *J* = 13.2 Hz, 7.5 Hz, 2.5 Hz, 1H), 7.71 (d, *J* = 9.0 Hz, 1H), 7.53 (m, 4H); ^**13**^**C NMR** (126 MHz, DMSO-*d*_*6*_) *δ* 160.7, 157.5, 150.3, 150.2, 137.1, 135.7, 135.6, 131.0, 128.5, 128.0, 125.4, 122.9, 119.0, 118.7, 117.3, 117.2, 117.0, 111.4 (d, *J* = 21.5 Hz); **HRMS** (ESI-MS): Calculated for C_20_H_13_N_4_O_2_F_2_ [M+H]^+^: 379.10011; found: 379.1013.

#### Preparation of *N*-(3,4-difluorobenzyl)-7-nitro-2-phenylquinazolin-4-amine (8f)

According to the general procedure, quinazoline **7a** (150 mg, 0.56 mmol) was converted and the crude product purified by silica gel column chromatography (2% → 20% EtOAc/PE) to the substituted aminoquinazoline **8f** (141 mg, 64%). ^**1**^**H NMR** (500 MHz, DMSO-*d*_*6*_) *δ* 9.30 (t, J = 5.7 Hz, 1H), 8.53 (d, *J* = 9.0, 1H), 8.46 (m, 3H), 8.23 (dd, *J* = 9.0 Hz, 2.3 Hz, 1H), 7.52 (m, 4H), 7.40 (dt, *J* = 10.7 Hz, 8.4 Hz, 1H), 7.33 (m, 1H), 4.90 (d, *J* = 5.6 Hz, 2H); ^**13**^**C NMR** (126 MHz, DMSO-*d*_*6*_) *δ* 161.0, 159.3, 150.1, 150.1, 148.2, 137.6, 137.0, 130.7, 128.3, 128.1, 125.2, 124.1, 122.8, 118.6, 117.4, 117.2, 116.5, 116.4, 43.2; **HRMS** (ESI-MS): Calculated for C_21_H_15_N_4_O_2_F_2_ [M+H]^+^: 393.11576; found: 393.11448.

#### Preparation of 7-nitro-2-phenyl-*N*-(thiophen-2-ylmethyl)quinazolin-4-amine (8g)

According to the general procedure, quinazoline **7a** (150 mg, 0.56 mmol) was converted and the crude product purified by silica gel column chromatography (1% → 20% EtOAc/PE) to the substituted aminoquinazoline **8g** (144 mg, 71%). ^**1**^**H NMR** (400 MHz, DMSO-*d*_*6*_) *δ* 9.52 (s, 1H), 8.58 (d, *J* = 3.5 Hz, 2H), 8.52 (d, *J* = 8.9 Hz, 2H), 8.24 (dd, *J* = 8.7 Hz, 2.0 Hz, 1H), 7.56 (s, 3H), 7.38 (d, *J* = 4.8 Hz, 1H), 7.20 (d, *J* = 2.7 Hz, 1H), 6.99 (m, 1H), 5.09 (d, *J* = 5.1 Hz, 2H); ^**13**^**C NMR** (101 MHz, DMSO-*d*_*6*_) *δ* 161.7, 159.8, 151.1, 142.4, 131.8, 129.3, 129.2, 127.4, 127.2, 126.4, 126.1, 123.5, 119.7, 118.2, 40.0; **HRMS** (ESI-MS): Calculated for C_19_H_15_N_4_O_2_S [M+H]^+^: 363.09102; found: 363.09115.

#### Preparation of 7-nitro-2-phenyl-*N*-(2-(thiophen-2-yl)ethyl)quinazolin-4-amine (8h)

According to the general procedure, quinazoline **7a** (150 mg, 0.56 mmol) was converted and the crude product purified by silica gel column chromatography (10% → 20% EtOAc/PE) to the substituted aminoquinazoline **8h** (137 mg, 65%). ^**1**^**H NMR** (500 MHz, DMSO-*d*_*6*_) *δ* 8.92 (s, 1H), 8.52 (dd, *J* = 6.6 Hz, 3.1 Hz, 2H), 8.48 (t, *J* = 5.3 Hz, 2H), 8.22 (dd, *J* = 8.9 Hz, 2.4 Hz, 1H), 7.53 (m, 3H), 7.34 (dd, *J* = 4.9 Hz, 1.3 Hz, 1H), 6.97 (dd, *J* = 8.3 Hz, 3.3 Hz, 2H), 3.94 (dd, *J* = 12.8 Hz, 7.0 Hz, 2H), 3.28 (s, 2H); ^**13**^**C NMR** (126 MHz, DMSO-*d*_*6*_) *δ* 161.1, 159.3, 150.1, 141.4, 139.2, 137.7, 130.7, 128.3, 128.1, 127.0, 125.3, 125.1, 124.2, 122.8, 118.5, 117.4, 42.6, 28.3; **HRMS** (ESI-MS): Calculated for C_20_H_17_N_4_O_2_S [M+H]^+^: 377.10667; found: 377.10703.

#### Preparation of 4-(2-(4-fluorophenyl)-4,5-dihydro-1*H*-imidazol-1-yl)-7-nitro-2-phenylquinazoline (8i)

According to the general procedure, quinazoline **7a** (150 mg, 0.56 mmol) was converted and the crude product purified by silica gel column chromatography (20% EtOAc/PE → 100% EtOAc) to the substituted aminoquinazoline **8i** (137 mg, 65%). In deviation from the common protocol K_2_CO_3_ (5eq, 323 mg, 2.34 mmol) was used as a base. ^**1**^**H NMR** (500 MHz, DMSO-*d*_*6*_) *δ* 8.66 (m, 1H), 8.54 (m, 1H), 8.31 (m, 1H), 7.72 (m, 4H), 7.44 (m, 1H), 7.32 (m, 2H), 7.23 (m, 2H), 4.49 (t, *J* = 8.4 Hz, 2H), 4.12 (t, *J* = 8.4 Hz, 2H); ^**13**^**C NMR** (126 MHz, DMSO-*d*_*6*_) *δ* 162.3, 161.6, 160.3, 153.0, 151.4, 137.2, 132.1, 131.0, 130.9, 129.8, 129.2, 128.9, 128.7, 124.1, 120.3, 120.2, 116.1 (d, *J* = 22.0 Hz), 56.4, 54.1; **HRMS** (ESI-MS): Calculated for C_20_H_17_N_4_O_2_S [M+H]^+^: 414.13608; found: 414.13580.

#### Preparation of *N*-((4-(cyclopropylmethyl)furan-2-yl)methyl)-7-nitro-2-phenylquinazolin-4-amine (8j)

According to the general procedure, quinazoline **7a** (180 mg, 0.67 mmol) was converted and the crude product purified by silica gel column chromatography (25% EtOAc/PE → 50% EtOAc) to the substituted aminoquinazoline **8j** as a hydrochloride salt (233 mg, 87%).

^**1**^**H NMR** (600 MHz, DMSO-*d*_*6*_) *δ* 8.59 (d, *J* = 9.0 Hz, 1H), 8.56–8.52 (m, 3H), 8.27 (dd, *J* = 8.9, 2.0 Hz, 1H), 7.62–7.54 (m, 4H), 6.33 (d, *J* = 3.0 Hz, 1H), 6.05 (d, *J* = 3.1 Hz, 1H), 4.88 (d, *J* = 5.4 Hz, 2H), 2.60 (d, *J* = 7.1 Hz, 2H), 2.39–2.37 (m, 1H), 1.83–1.55 (m, 4H); ^**13**^**C NMR** (151 MHz, DMSO-*d*_*6*_) *δ* 159.2, 154.6, 150.3, 145.8, 132.0, 128.7, 128.5, 128.3, 128.1, 125.5, 120.1, 117.3, 111.1, 108.4, 106.4, 106.0, 45.5, 31.9, 27.0, 25.3; **HRMS** (ESI-MS): Calculated for C_23_H_22_N_4_O_3_Cl [M+H]^+^: 437.13749 and 439.13454; found: 437.13815 and 439.13526.

#### Preparation of 8-nitro-*N*-phenethyl-2-phenylquinazolin-4-amine (8k)

According to the general procedure, quinazoline **7b** (268 mg, 1.00 mmol) was converted and the crude product purified by silica gel column chromatography (30% EtOAc/PE) to the substituted aminoquinazoline **8k** (324 mg, 87%). ^**1**^**H NMR** (500 MHz, DMSO-*d*_*6*_) *δ* 8.86 (t, *J* = 5.4 Hz, 1H), 8.49 (d, *J* = 8.3 Hz, 1H), 8.46–8.42 (m, 2H), 8.24 (d, *J* = 7.6 Hz, 1H), 7.60 (t, *J* = 7.9 Hz, 1H), 7.55–7.51 (m, 3H), 7.35–7.29 (m, 4H), 7.24–7.18 (m, 1H), 3.93 (dd, *J* = 14.1, 6.3 Hz, 2H), 3.08 (m, 2H); ^**13**^**C NMR** (101 MHz, DMSO-*d*_*6*_) *δ* 161.0, 159.2, 147.0, 141.9, 139.4, 137.7, 131.0, 128.8, 128.5, 128.5, 128.2, 126.7, 126.5, 126.2, 124.2, 114.9, 42.6, 34.4; **HRMS** (ESI-MS): Calculated for C_22_H_19_N_4_O_2_ [M+H]^+^: 371.15025; found: 371.15018.

#### Preparation of 1-(4-(4-(7-nitro-4-(phenethylamino)quinazolin-2-yl)phenyl)piperazin-1-yl)ethan-1-one (8l)

According to the general procedure, quinazoline **3c** (366 mg, 0.74 mmol) was converted and the crude product purified by flash chromatography (0% → 10% MeOH/DCM) to the substituted aminoquinazoline **8l** (329 mg, 69%). ^**1**^**H NMR** (500 MHz, DMSO-*d*_*6*_) *δ* 8.73 (t, J = 5.3 Hz, 1H), 8.47–8.33 (m, 4H), 8.12 (dd, J = 8.9, 2.3 Hz, 1H), 7.32 (d, J = 4.4 Hz, 4H), 7.27–7.17 (m, 1H), 7.07 (d, J = 9.0 Hz, 2H), 3.88 (dd, J = 14.3, 6.1 Hz, 2H), 3.61 (s, 4H), 3.34 (dd, J = 10.1, 5.3 Hz, 2H), 3.30–3.27 (m, 2H), 3.10–3.02 (m, 2H), 2.06 (s, 3H); ^**13**^**C NMR** (126 MHz, DMSO-*d*_*6*_) *δ* 168.3, 161.3, 159.0, 152.3, 150.8, 150.3, 150.0, 139.4, 129.4, 128.7,128.4, 127.8, 126.1, 125.0, 122.4, 117.5,117.2, 114.2, 47.4, 47.1, 45.2, 42.5, 40.5, 34.3, 21.1; **HRMS** (ESI-MS): Calculated for C_28_H_29_N_6_O_3_ [M+H]^+^: 497.22957; found: 497.22855.

#### Preparation of 2-(4-morpholinophenyl)-7-nitro-*N*-phenethylquinazolin-4-amine (8m)

According to the general procedure, quinazoline **7d** (490 mg, 1.22 mmol) was converted and the crude product purified by flash chromatography (0% → 10% MeOH/DCM) to the substituted aminoquinazoline **8m** (268 mg, 48%). ^**1**^**H NMR** (400 MHz, DMSO-*d*_*6*_) *δ* 8.89–8.71 (m, *J* = 5.0 Hz, 1H), 8.52–8.35 (m, 4H), 8.15 (dd, *J* = 8.9, 2.3 Hz, 1H), 7.33 (d, *J* = 4.3 Hz, 4H), 7.27–7.16 (m, *J* = 8.6, 4.3 Hz, 1H), 7.08 (d, *J* = 8.9 Hz, 2H), 3.96–3.84 (m, *J* = 13.0, 6.7 Hz, 2H), 3.77 (t, *J* = 4.0 Hz, 4H), 3.30–3.21 (m, *J* = 4.3 Hz, 4H), 3.06 (m, 2H); ^**13**^**C NMR** (126 MHz, DMSO-*d*_*6*_) *δ* 162.2, 158.5, 153.7, 150.9, 140.4, 130.3, 130.2, 129.8, 129.4, 129.1, 128.8, 127.0, 125.9, 123.3, 114.7, 66.9, 48.4, 43.4, 35.2; **HRMS** (ESI-MS): Calculated for C_26_H_26_N_5_O_3_ [M+H]^+^: 456.20302; found 456.20230.

#### Preparation of 2-(4-(methylsulfinyl)phenyl)-7-nitro-*N*-phenethylquinazolin-4-amine (18)

Following the general procedure, quinazoline **7e** (400 mg, 1.27 mmol) was converted and the crude product **8n** was subsequently used without any further purification. To a stirred solution of nitroquinazoline **8n** (500 mg, 1.20 mmol) in DCM (10 mL) *m*CPBA (800 mg, 4.80 mmol) was added in small portions at 0°C. The reaction mixture was allowed to warm up to rt and was stirred for 3 h. After quenching with saturated NaHCO_3_:NaS_2_O_3_ (1:1) and extraction, the combined organic layers were concentrated under reduced pressure. The described compound **18** was obtained after precipitation in cold H_2_O (478 mg, 87%). ^**1**^**H NMR** (500 MHz, DMSO-*d*_*6*_) *δ* 8.96 (t, *J* = 5.4 Hz, 1H), 8.68 (d, *J* = 8.5 Hz, 2H), 8.50–8.45 (m, 2H), 8.23 (dd, *J* = 8.9, 2.4 Hz, 1H), 8.09 (d, *J* = 8.5 Hz, 2H), 7.36–7.29 (m, 4H), 7.24–7.19 (m, 1H), 3.91 (dd, *J* = 14.1, 6.3 Hz, 2H), 3.06 (t, *J* = 7.5 Hz, 2H); ^**13**^**C NMR** (126 MHz, DMSO-*d*_*6*_) *δ* 159.7, 159.4, 150.1, 149.8, 142.4, 142.3, 139.3, 128.7, 128.4, 127.1, 126.2, 125.2, 122.9, 119.1, 117.6, 43.5, 42.6, 34.3; **HRMS** (ESI-MS): Calculated for C_23_H_21_N_4_O_3_S [M+H]^+^: 433.13289; found 433.13260.

### Common procedure for the generation of 7-/8-aminoquinazolines 9a-m

The corresponding nitroquinazoline was reduced with 10% Pd/C (0.2 mol-%) and ammonium formate (6 eq) in EtOH for 2 h at 80°C. The hot reaction mixture was filtered through Celite and concentrated under reduced pressure. Sulphur-containing nitroquinazolines were alternatively reduced using Fe (5 eq) and NH_4_Cl (8 eq) in MeOH:H_2_O (4:1) for 3–4 h at 80°C. The crude reaction mixture was partitioned between NaHCO_3_ and DCM and the combined organic layers were concentrated *in vacuo*.

#### Preparation of *N*^*4*^-benzyl-2-phenylquinazoline-4,7-diamine (9a)

According to the common procedure, quinazoline **8a** (100 mg, 0.30 mmol) was converted and the crude product was purified by silica gel column chromatography (1% → 10% MeOH/DCM) to yield the amine **9a** (90 mg, 93%). ^**1**^**H NMR** (500 MHz, DMSO-*d*_*6*_) *δ* 8.51 (s, *J* = 40.7 Hz, 1H), 8.40–8.33 (m, 2H), 7.98 (d, *J* = 12.2 Hz, 1H), 7.44 (m, 5H), 7.31 (t, *J* = 7.5 Hz, 2H), 7.22 (t, *J* = 7.3 Hz, 1H), 6.80 (dd, *J* = 8.8, 1.9 Hz, 1H), 6.78–6.75 (m, *J* = 1.9 Hz, 1H), 5.94 (s, 2H), 4.87 (d, *J* = 5.7 Hz, 2H); ^**13**^**C NMR** (126 MHz, DMSO-*d*_*6*_) *δ* 159.0, 158.8, 152.9, 140.3, 129.8, 128.2, 128.1, 127.7, 127.4, 126.6, 123.7, 116.0, 105.6, 104.3, 43.6; **HRMS** (ESI-MS): Calculated for C_21_H_19_N_4_ [M+H]^+^: 327.16042; found 327.16056.

#### Preparation of *N*-(4-fluorophenyl)-7-nitro-2-phenylquinazolin-4-amine (9b)

Following the common procedure, quinazoline **8b** (513 mg, 1.42 mmol) was converted and the crude product was purified by silica gel column chromatography (2% MeOH/DCM) to yield the amine **9b** (264 mg, 56%). ^**1**^**H NMR** (300 MHz, DMSO-*d*_*6*_) *δ* 9.48 (s, 1H), 8.43–8.29 (m, 2H), 8.19 (d, *J* = 9.0 Hz, 1H), 8.01–7.82 (m, 2H), 7.55–7.39 (m, 3H), 7.35–7.19 (m, 2H), 6.90 (dd, *J* = 8.9, 2.1 Hz, 1H), 6.81 (d, *J* = 2.1 Hz, 1H), 6.06 (s, 2H); ^**13**^**C NMR** (75 MHz, DMSO-*d*_*6*_) *δ* 158.8, 158.0 (d, *J* = 239.6 Hz), 157.2, 153.2, 152.5, 138.7, 136.3, 129.9, 128.3, 127.7, 124.0, 123.6 (d, *J* = 7.8 Hz), 116.4, 115.0 (d, *J* = 22.1 Hz), 106.0, 104.6; **HRMS** (ESI-MS): Calculated for C_20_H_16_N_4_F [M+H]^+^: 331.13535; found 331.13574.

#### Preparation of *N*^*4*^-(4-fluorobenzyl)-2-phenylquinazoline-4,7-diamine (9c)

According to the common procedure, quinazoline **8c** (280 mg, 0.75 mmol) was converted and the crude product was purified by silica gel column chromatography (0.5% → 2% MeOH/DCM) to yield the amine **9c** (157 mg, 61%). ^**1**^**H NMR** (300 MHz, DMSO-*d*_*6*_) *δ* 8.46 (m, 1H), 8.42–8.34 (m, 2H), 7.95 (d, *J* = 8.8 Hz, 1H), 7.59–7.29 (m, 5H), 7.14 (t, *J* = 8.9 Hz, 2H), 6.79 (dd, *J* = 8.8, 2.2 Hz, 1H), 6.74 (d, *J* = 2.1 Hz, 1H), 5.93 (s, 2H), 4.83 (d, *J* = 5.7 Hz, 2H); ^**13**^**C NMR** (75 MHz, DMSO-*d*_*6*_) *δ* 161.1 (d, *J* = 242.0 Hz), 159.0, 158.9, 152.9, 138.8, 136.6, 129.8, 129.3 (d, *J* = 8.1 Hz), 128.1, 127.7, 123.7, 115.7, 115.0 (d, *J* = 21.2 Hz), 105.9, 104.5, 42.9; **HRMS** (ESI-MS): Calculated for C_21_H_18_N_4_F [M+H]^+^: 345.15100; found 345.15117.

#### Preparation of *N*^*4*^-(3-fluorophenyl)-2-phenylquinazoline-4,7-diamine (9d)

Following the common procedure, quinazoline **8d** (68 mg, 0.19 mmol) was converted and the crude product was purified by silica gel column chromatography (1% → 5% MeOH/DCM) to yield the amine **9d** (60 mg, 96%). ^**1**^**H NMR** (300 MHz, DMSO-*d*_*6*_) *δ* 9.67 (s, 1H), 8.45–8.32 (m, 2H), 8.24 (d, *J* = 9.0 Hz, 1H), 8.01 (d, *J* = 12.2 Hz, 1H), 7.74 (d, *J* = 8.4 Hz, 1H), 7.60–7.47 (m, 3H), 7.46–7.39 (m, 1H), 6.92 (td, *J* = 8.7, 2.2 Hz, 2H), 6.85 (d, *J* = 2.0 Hz, 1H), 6.19 (s, 2H); ^**13**^**C NMR** (75 MHz, DMSO-*d*_*6*_) *δ* 162.1 (d, *J* = 240.3 Hz), 158.6, 157.1, 153.5, 141.8 (d, *J* = 10.8 Hz), 130.1 (d, *J* = 18.66 Hz), 129.9, 128.4, 127.8, 124.2, 117.1, 116.7, 109.1 (d, *J* = 19.4 Hz), 108.1 (d, *J* = 25.4 Hz), 104.6; **HRMS** (ESI-MS): Calculated for C_20_H_16_N_4_F [M+H]^+^: 331.13535; found 331.13580.

#### Preparation of *N*^*4*^-(3,4-difluorophenyl)-2-phenylquinazoline-4,7-diamine (9e)

According to the common procedure, quinazoline **8e** (59 mg, 0.16 mmol) was converted and the crude product was purified by silica gel column chromatography (0% → 1% MeOH/DCM) to yield the amine **9e** (36 mg, 66%). ^**1**^**H NMR** (400.1 MHz, DMSO-*d*_*6*_) *δ* 9.57 (s, 1H), 8.38 (d, *J* = 7.8 Hz, 2H), 8.20 (d, *J* = 8.8 Hz, 2H), 7.71 (m, 1H), 7.48 (m, 4H), 6.92 (dd, *J* = 8.9 Hz, 1H), 6.83 (s, 1H), 6.11 (s, 2H); ^**13**^**C NMR** (100.6 MHz, DMSO-*d*_*6*_) *δ* 159.6, 157.7, 154.1, 163.6, 139.6, 138.1, 138.0, 130.8, 129.2, 128.5, 124.7, 118.3, 117.9, 117.8, 117.4, 111.1 (d, *J* = 21.6 Hz), 107.0, 105.5; **HRMS** (ESI-MS): Calculated for C_20_H_15_N_4_F_2_ [M+H]^+^: 349.12593; found 349.12594.

#### Preparation of *N*^*4*^-(3,4-difluorobenzyl)-2-phenylquinazoline-4,7-diamine (9f)

Following the common procedure, quinazoline **8f** (53 mg, 0.13 mmol) was converted and the crude product was purified by silica gel column chromatography (0% → 10% MeOH/DCM) to yield the amine **9f** (35 mg, 72%). ^**1**^**H NMR** (500 MHz, DMSO-*d*_*6*_) *δ* 8.49 (s, 1H), 8.36 (dd, *J* = 6.6 Hz, 3.0 Hz, 2H), 8.14 (s, 1H), 7.95 (d, *J* = 8.8 Hz, 1H), 7.45 (m, 4H), 7.37 (dd, *J* = 19.2 Hz, 8.5 Hz, 1H), 7.28 (m, 1H), 6.82 (dd, *J* = 8.8 Hz, 1.7 Hz, 1H), 6.77 (s, 1H), 5.94 (s, 2H), 4.82 (d, *J* = 5.7 Hz, 2H); ^**13**^**C NMR** (101 MHz, DMSO-*d*_*6*_) *δ* 163.2, 158.9, 158.8, 153.0, 138.5, 138.4, 129.9, 128.2, 127.8, 124.0, 123.8, 117.4, 117.2, 116.4, 116.2, 115.9, 105.6, 104.3, 42.8; **HRMS** (ESI-MS): Calculated for C_21_H_17_N_4_F_2_ [M+H]^+^: 363.14158; found: 363.14154.

#### Preparation of 2-phenyl-*N*^*4*^-(thiophen-2-ylmethyl)quinazoline-4,7-diamine (9g)

According to the common procedure, quinazoline **8g** (59 mg, 0.16 mmol) was converted and the crude product was purified by silica gel column chromatography (1% → 10% MeOH/DCM) to yield the amine **9g** (45 mg, 83%). ^**1**^**H NMR** (500 MHz, DMSO-*d*_*6*_) *δ* 8.97 (s, 1H), 8.47 (dd, *J* = 6.6 Hz, 3.0 Hz, 2H), 7.98 (d, *J* = 9.4 Hz, 1H), 7.53 (m, 3H), 7.35 (dd, *J* = 5.1 Hz, 0.9 Hz, 1H), 7.13 (d, *J* = 2.9 Hz, 1H), 6.96 (dd, *J* = 5.0 Hz, 3.5 Hz, 1H), 6.83 (d, *J* = 6.6 Hz, 2H), 6.19 (s, 2H), 5.02 (d, *J* = 5.8 Hz, 2H); ^**13**^**C NMR** (101 MHz, DMSO-*d*_*6*_) *δ* 159.4, 158.9, 154.6, 143.3, 131.7, 129.2, 127.5, 127.3, 126.2, 125.1, 117.0, 104.3, 49.45; **HRMS** (ESI-MS): Calculated for C_19_H_17_N_4_S [M+H]^+^: 333.11684; found: 333.11689.

#### Preparation of 2-phenyl-*N*^*4*^-(2-(thiophen-2-yl)ethyl)quinazoline-4,7-diamine (9h)

Following the common procedure, quinazoline **8h** (60 mg, 0.16 mmol) was converted and the crude product was purified by silica gel column chromatography (0% → 2% MeOH/DCM) to yield the amine **9h** (46 mg, 84%). ^**1**^**H NMR** (500 MHz, DMSO-*d*_*6*_) *δ* 9.76 (s, 1H), 8.33 (m, 2H), 8.17 (d, *J* = 9.1 Hz, 1H), 7.72 (t, *J* = 7.3 Hz, 1H), 7.66 (t, *J* = 7.5 Hz, 2H), 7.34 (dd, *J* = 5.0 Hz, 1.2 Hz, 1H), 7.03 (d, *J* = 2.1 Hz, 1H), 6.95 (m, 5H), 3.97 (dd, *J* = 12.9 Hz, 7.0 Hz, 2H), 3.27 (t, *J* = 7.2, 2H); ^**13**^**C NMR** (126 MHz, DMSO-*d*_*6*_) *δ* 158.8, 156.4, 155.3, 140.8, 133.0, 128.8, 128.7, 127.0, 125.5, 125.4, 124.3, 117.0, 101.2, 42.8; **HRMS** (ESI-MS): Calculated for C_20_H_19_N_4_S [M+H]^+^: 347.13249; found: 347.13256.

#### Preparation of 4-(2-(4-fluorophenyl)-4,5-dihydro-1*H*-imidazol-1-yl)-2-phenylquinazolin-7-amine (9i)

According to the common procedure, quinazoline **8i** (80 mg, 0.20 mmol) was converted and the crude product was purified by silica gel column chromatography (0% → 1% MeOH/DCM) to yield the amine **9i** (61 mg, 81%). ^**1**^**H NMR** (500 MHz, DMSO-*d*_*6*_) *δ* 7.91 (m, 1H), 7.72 (d, *J* = 7.3 Hz, 2H), 7.61 (dd, *J* = 8.6 Hz, 5.6 Hz, 2H), 7.35 (t, *J* = 7.3 Hz, 1H), 7.27 (dd, *J* = 14.3 Hz, 6.6 Hz, 2H), 7.17 (t, *J* = 8.8 Hz, 2H), 6.99 (dd, *J* = 9.0 Hz, 2.1 Hz, 1H), 6.84 (d, *J* = 2.1 Hz, 1H), 6.30 (s, 2H), 4.27 (t, *J* = 8.7 Hz, 2H), 4.04 (t, *J* = 8.7 Hz, 2H); ^**13**^**C NMR** (126 MHz, DMSO-*d*_*6*_) *δ* 163.6, 161.7, 160.7, 157.8, 154.5, 153.8, 137.8, 129.9, 129.7, 129.7, 127.9, 127.3, 126.2, 117.7, 115.1, 115.0, 114.9, 114.8, 107.9, 105.1, 55.1, 53.3; **HRMS** (ESI-MS): Calculated for C_23_H_19_N_5_F [M+H]^+^: 384.16190; found: 384.16173.

#### Preparation of *N*^*4*^-((4-(cyclopropylmethyl)furan-2-yl)methyl)-2-phenylquinazoline-4,7-diamine (9j)

Following the common procedure, quinazoline **8j** (135 mg, 0.31 mmol) was converted and the crude product was purified by silica gel column chromatography (0.5% → 5% MeOH/DCM) to yield the amine **9j** as a hydrochloride salt (99 mg, 87%). ^**1**^**H NMR** (600 MHz, DMSO-*d*_*6*_) *δ* 8.46 (dd, *J* = 17.5, 6.8 Hz, 2H), 7.95 (dd, *J* = 25.3, 8.7 Hz, 1H), 7.58–7.39 (m, 3H), 6.77 (d, *J* = 8.7 Hz, 1H), 6.74 (s, 1H), 6.22 (d, *J* = 2.6 Hz, 1H), 6.01 (d, *J* = 3.0 Hz, 1H), 5.87 (br, 1H), 4.76 (d, *J* = 5.0 Hz, 2H), 3.63–3.57 (m, 2H), 2.59 (d, *J* = 7.1 Hz, 2H), 2.40–2.37 (m, 1H), 1.79–1.62 (m, 4H); ^**13**^**C NMR** (151 MHz, DMSO-*d*_*6*_) *δ* 159.3, 154.6, 151.9, 139.8, 130.1, 128.6, 128.3, 124.2, 116.2, 108.0, 106.3, 45.5, 40.5, 31.9, 27.1, 25.3; **HRMS** (ESI-MS): Calculated for C_23_H_24_N_4_OCl [M+H]^+^: 407.16332 and 409.16037; found: 407.16399 and 409.16100.

#### Preparation of *N*^*4*^-phenethyl-2-phenylquinazoline-4,8-diamine (9k)

Following the common procedure, quinazoline **8k** (370 mg, 1.00 mmol) was converted and the crude product was purified by silica gel column chromatography (25% → 35% EtOAc/PE) to yield the amine **9k** (271 mg, 80%). ^**1**^**H NMR** (500 MHz, DMSO-*d*_*6*_) *δ* 8.59 (d, *J* = 8.2 Hz, 2H), 8.11 (t, *J* = 5.2 Hz, 1H), 7.55–7.42 (m, 3H), 7.35–7.30 (m, 4H), 7.29 (d, *J* = 8.4 Hz, 1H), 7.24–7.18 (m, 1H), 7.16 (t, *J* = 7.9 Hz, 1H), 6.90 (d, *J* = 7.6 Hz, 1H), 5.88 (s, 2H), 3.87 (dd, *J* = 13.7, 6.7 Hz, 2H), 3.06 (t, *J* = 7.5 Hz, 2H); ^**13**^**C NMR** (126 MHz, DMSO-*d*_*6*_) *δ* 159.5, 156.4, 144.8, 139.7, 138.9, 138.4, 129.6, 128.7, 128.4, 128.1, 127.7, 126.0, 125.8, 113.8, 112.0, 108.0, 42.4, 34.7; **HRMS** (ESI-MS): Calculated for C_22_H_21_N_4_ [M+H]^+^: 341.17607; found: 341.17608.

#### Preparation of 1-(4-(4-(7-amino-4-(phenethylamino)quinazolin-2-yl)phenyl)piperazin-1-yl)ethan-1-one (9l)

According to the common procedure, quinazoline **8l** (268 mg, 0.54 mmol) was converted and the crude product was purified by flash chromatography (2% → 10% MeOH/DCM) to yield the amine **9l** (147 mg, 58%). ^**1**^**H NMR** (500 MHz, DMSO-*d*_*6*_) *δ* 13.26 (s, 1H), 9.60 (t, J = 5.4 Hz, 1H), 8.32 (t, J = 15.3 Hz, 2H), 8.14 (d, J = 9.1 Hz, 1H), 7.30 (d, J = 4.4 Hz, 4H), 7.23–7.17 (m, 1H), 7.11 (d, J = 9.1 Hz, 2H), 7.05 (d, J = 1.9 Hz, 1H), 6.86 (dd, J = 9.0, 2.0 Hz, 1H), 6.79 (s, 1H), 3.91 (dt, J = 13.9, 6.4 Hz, 2H), 3.64–3.58 (m, 4H), 3.47 (t, J = 4.9 Hz, 2H), 3.40 (s, 2H), 3.03 (t, J = 7.5 Hz, 2H), 2.06 (s, 3H); ^**13**^**C NMR** (126 MHz, DMSO-*d*_*6*_) *δ* 168.4, 158.5, 155.7, 155.1, 153.5, 141.2, 139.0, 130.4, 128.7, 128.4, 126.2, 125.3, 119.2, 116.0, 113.4, 100.9, 97.4, 46.5, 46.2, 44.9, 42.6, 40.3, 34.8, 21.2; **HRMS** (ESI-MS): Calculated for C_28_H_31_N_6_O [M+H]^+^: 467.25539; found: 467.25443.

#### Preparation of 2-(4-morpholinophenyl)-*N*^*4*^-phenethylquinazoline-4,7-diamine (9m)

Following the common procedure, quinazoline **8m** (21 mg, 0.05 mmol) was converted and the crude product was purified by flash chromatography (2% → 10% MeOH/DCM) to yield the amine **9m** (13 mg, 66%). ^**1**^**H NMR** (400 MHz, CDCl_3_) *δ* 8.46 (d, *J* = 8.9 Hz, 2H), 7.27 (d, *J* = 9.7 Hz, 6H), 6.98 (dd, *J* = 6.7, 4.2 Hz, 3H), 6.67 (dd, *J* = 8.7, 2.2 Hz, 1H), 5.93 (s, 1H), 4.11 (br, 2H), 3.97 (dd, *J* = 12.9, 6.9 Hz, 2H), 3.92–3.86 (m, 4H), 3.31–3.24 (m, 4H), 3.06 (t, *J* = 7.1 Hz, 2H); ^**13**^**C NMR** (101 MHz, CDCl_3_) *δ* 159.0, 152.8, 150.5, 143.5, 139.6, 129.8, 129.1, 128.8, 126.6, 122.4, 115.4, 114.6, 77.4, 67.0, 48.7, 42.5, 35.7; **HRMS** (ESI-MS): Calculated for C_26_H_28_N_5_O [M+H]^+^: 426.22884; found: 426.22857.

#### Preparation of 2-(4-(methylsulfinyl)phenyl)-*N*^*4*^-phenethylquinazoline-4,7-diamine (19)

According to the common procedure, quinazoline **18** (478 mg, 1.11 mmol) was converted and the crude product was purified by flash chromatography (0% → 6% MeOH/DCM) to yield the amine **19** (272 mg, 61%). ^**1**^**H NMR** (500 MHz, CDCl_3_) *δ* 8.73 (d, *J* = 8.4 Hz, 2H), 8.03 (d, *J* = 8.4 Hz, 2H), 7.41–7.30 (m, 4H), 7.28 (d, *J* = 7.7 Hz, 3H), 7.03 (d, *J* = 2.0 Hz, 1H), 6.79 (dd, *J* = 8.7, 2.0 Hz, 1H), 5.61 (s, 1H), 4.17 (s, 2H), 3.07 (s, 3H), 3.06 (s, 2H); ^**13**^**C NMR** (126 MHz, CDCl_3_) *δ* 159.4, 159.2, 152.3, 150.7, 144.8, 141.1, 139.3, 129.3, 129.0, 128.9, 127.3, 126.8, 122.2, 116.7, 109.4, 106.7, 44.8, 42.5, 35.7; **HRMS** (ESI-MS): Calculated for C_23_H_23_N_4_OS [M+H]^+^: 403.15871; found: 403.15755.

#### Preparation of *N*-(4-(phenethylamino)-2-phenylquinazolin-7-yl)propionamide (10a)

To a stirred solution of **3** (40 mg, 0.12 mmol) in DIPEA (40 μL, 0.24 mmol) and THF (5 mL) at 0°C propionyl chloride (12.3 μL in 1 mL THF, 0.14 mmol) was added dropwise. The reaction mixture was allowed to warm up to rt and was stirred for another 2 h. After extraction with saturated NaHCO_3_, concentration of the combined organic layers *in vacuo* and silica gel column chromatography (0% → 1% MeOH/DCM) **10a** (31 mg, 66%) was obtained. ^**1**^**H NMR** (500 MHz, DMSO-*d*_*6*_) *δ* 10.19 (s, 1H), 8.50 (dd, *J* = 8.0, 1.6 Hz, 2H), 8.29 (t, *J* = 5.4 Hz, 1H), 8.13 (dd, *J* = 5.4, 3.2 Hz, 2H), 7.58 (dd, *J* = 8.9, 2.0 Hz, 1H), 7.54–7.45 (m, 3H), 7.36–7.29 (m, 4H), 7.25–7.18 (m, 1H), 3.86 (dt, *J* = 14.5, 6.0 Hz, 2H), 3.06 (m, 2H), 2.41 (q, *J* = 7.5 Hz, 2H), 1.13 (t, *J* = 7.5 Hz, 3H); ^**13**^**C NMR** (126 MHz, DMSO-*d*_*6*_) *δ* 172.6, 159.6, 159.1, 151.0, 142.9, 139.7, 138.8, 129.9, 128.7, 128.4, 128.2, 127.8, 126.1, 123.2, 117.7, 114.9, 109.5, 42.3, 34.7, 29.7, 9.5; **HRMS** (ESI-MS): Calculated for C_25_H_25_N_4_O [M+H]^+^: 397.20229; found: 397.20133.

#### Preparation of *N*-(4-(phenethylamino)-2-phenylquinazolin-7-yl)propane-1-sulfonamide (10b)

To a stirred solution of **3** (75 mg, 0.22 mmol) in pyridine (19.6 μL, 0.24 mmol) and DCM (5 mL) at 0°C propane-1-sulfonyl chloride (27.2 μL in 1 mL DCM, 0.24 mmol) was added dropwise. The reaction mixture was then heated to 50°C and was stirred for another 6 h. After quenching with 6 N NaOH and extraction, the combined organic layers were concentrated *in vacuo*. Silica gel column chromatography (0.5% MeOH/DCM) yielded **10b** (68 mg, 80%). ^**1**^**H NMR** (500 MHz, DMSO-*d*_*6*_) *δ* 10.31 (s, 1H), 8.49 (dd, *J* = 7.7, 1.8 Hz, 2H), 8.36 (s, 1H), 8.16 (d, *J* = 8.9 Hz, 1H), 7.58–7.43 (m, 4H), 7.34–7.31 (m, 4H), 7.29 (dd, *J* = 8.9, 2.1 Hz, 1H), 7.25–7.18 (m, 1H), 3.87 (dd, *J* = 14.3, 6.1 Hz, 2H), 3.22 (m, 2H), 3.05 (m, 2H), 1.79–1.67 (m, 2H), 0.95 (t, *J* = 7.5 Hz, 3H); ^**13**^**C NMR** (101 MHz, DMSO-*d*_*6*_) *δ* 160.0, 159.3, 151.1, 142.4, 139.7, 138.6, 130.2, 128.8, 128.5, 128.3, 127.9, 126.2, 124.2, 117.0, 113.7, 109.7, 52.7, 42.4, 34.7, 17.0, 12.5; **HRMS** (ESI-MS): Calculated for C_25_H_27_N_4_O_2_S [M+H]^+^: 447.18492; found: 447.18460.

#### Preparation of *N*-(4-(phenethylamino)-2-phenylquinazolin-7-yl)benzenesulfonamide (10c)

To a stirred solution of **3** (75 mg, 0.22 mmol) in pyridine (19.6 μL, 0.24 mmol) and DCM (5 mL) at 0°C benzenesulfonyl chloride (31 μL in 1 mL DCM, 0.24 mmol) was added dropwise. The reaction mixture was then heated to 50°C and was stirred for another 6 h. After quenching with 6 N NaOH and extraction, the combined organic layers were concentrated *in vacuo*. Silica gel column chromatography (30% → 35% EtOAc/PE) yielded **10c** (32 mg, 69%). ^**1**^**H NMR** (500 MHz, DMSO-*d*_*6*_) *δ* 10.87 (s, 1H), 8.47–8.41 (m, 2H), 8.08 (d, *J* = 8.9 Hz, 1H), 7.89–7.85 (m, 2H), 7.65–7.54 (m, 3H), 7.53–7.45 (m, 3H), 7.41 (d, *J* = 1.8 Hz, 1H), 7.35–7.27 (m, 4H), 7.26–7.17 (m, 2H), 5.74 (s, 1H), 3.83 (dd, *J* = 14.5, 6.1 Hz, 2H), 3.01 (m, 2H); ^**13**^**C NMR** (101 MHz, DMSO-*d*_*6*_) *δ* 159.9, 159.2, 154.5, 147.2, 141.7, 139.6, 139.3, 133.3, 130.3, 129.5, 128.7, 128.5, 128.3, 127.9, 126.7, 126.2, 124.1, 117.4, 114.6, 110.0, 42.4, 34.7; **HRMS** (ESI-MS): Calculated for C_28_H_25_N_4_O_2_S [M+H]^+^: 481.16927; found: 481.16906.

#### Preparation of *N*-(4-(phenethylamino)-2-phenylquinazolin-8-yl)propane-1-sulfonamide (10d)

To a stirred solution of **9k** (60 mg, 0.18 mmol) in pyridine (2 drops) and DCM (5 mL) at 0°C propane-1-sulfonyl chloride (23.4 μL in 1 mL DCM, 0.19 mmol) was added dropwise. The reaction mixture was then heated to 50°C and was stirred for another 6 h. After quenching with 6 N NaOH and extraction, the combined organic layers were concentrated *in vacuo*. Silica gel column chromatography (10% → 20% EtOAc/PE) yielded **10b** (65 mg, 83%). ^**1**^**H NMR** (500 MHz, DMSO-*d*_*6*_) *δ* 9.24 (s, 1H), 8.63 (dd, *J* = 7.8, 1.5 Hz, 2H), 8.57 (t, *J* = 5.4 Hz, 1H), 7.97 (d, *J* = 8.3 Hz, 1H), 7.76 (d, *J* = 7.6 Hz, 1H), 7.58–7.48 (m, 3H), 7.45 (t, *J* = 8.0 Hz, 1H), 7.36–7.28 (m, 4H), 7.24–7.17 (m, 1H), 3.90 (dd, *J* = 14.3, 6.2 Hz, 2H), 3.23–3.15 (m, 2H), 3.10–3.04 (m, 2H), 1.82–1.71 (m, 2H), 0.85 (t, *J* = 7.4 Hz, 2H); ^**13**^**C NMR** (126 MHz, DMSO-*d*_*6*_) *δ* 159.5, 158.8, 141.4, 139.5, 138.2, 133.5, 130.3, 128.7, 128.4, 128.2, 128.2, 126.1, 125.0, 121.0, 117.6, 113.8, 53.0, 42.5, 34.5, 16.8, 12.5; **HRMS** (ESI-MS): Calculated for C_25_H_27_N_4_O_2_S [M+H]^+^: 447.18492; found: 447.18435.

#### Preparation of *N*-(4-(phenethylamino)-2-phenylquinazolin-8-yl)benzenesulfonamide (10e)

To a stirred solution of **3** (60 mg, 0.18 mmol) in pyridine (2 drops) and DCM (5 mL) at 0°C benzenesulfonyl chloride (26.7 μL in 1 mL DCM, 0.19 mmol) was added dropwise. The reaction mixture was then heated to 50°C and was stirred for another 6 h. After quenching with 6 N NaOH and extraction, the combined organic layers were concentrated *in vacuo*. Silica gel column chromatography (10% → 20% EtOAc/PE) yielded **10c** (41 mg, 78%). ^**1**^**H NMR** (300 MHz, DMSO-*d*_*6*_) *δ* 9.87 (s, 1H), 8.60 (m, 2H), 8.50 (t, *J* = 5.4 Hz, 1H), 7.95–7.84 (m, 3H), 7.71 (d, *J* = 7.1 Hz, 1H), 7.60–7.46 (m, 4H), 7.47–7.34 (m, 3H), 7.34–7.24 (m, 4H), 7.24–7.15 (m, 1H), 3.84 (dd, *J* = 14.3, 6.1 Hz, 2H), 3.02 (m, 2H); ^**13**^**C NMR** (75 MHz, DMSO-*d*_*6*_) *δ* 159.4, 158.7, 141.6, 139.6, 138.2, 133.0, 132.8, 130.4, 129.1, 128.7, 128.5, 128.4, 128.2, 126.8, 126.2, 124.8, 121.3, 118.0, 113.7, 42.5, 34.5; **HRMS** (ESI-MS): Calculated for C_28_H_25_N_4_O_2_S [M+H]^+^: 481.16927; found: 481.16884.

#### Preparation of *N*-(furan-2-ylmethyl)acetamide (12)

To a solution of furan-2-ylmethanamine **11** (920 μL, 10.4 mmol) in DCM (50 mL) Ac_2_O (2.9 mL, 31 mmol) and TEA (2.86 g, 20.6 mmol) were added and the reaction mixture was stirred overnight at rt. The solvent was removed under reduced pressure and after silica gel column chromatography (5% → 50% EtOAc/PE) **12** (1.26 g, 88%) was obtained. ^**1**^**H NMR** (300 MHz, DMSO-*d*_*6*_) *δ* 8.30 (s, 1H), 7.56 (d, J = 1.0 Hz, 1H), 6.38 (dd, J = 3.1 Hz, 1.9 Hz, 1H), 6.22 (d, J = 2.8 Hz, 1H) 4.23 (d, J = 5.7 Hz, 2H), 1.83 (s, 3H); ^**13**^**C NMR** (126 MHz, DMSO-*d*_*6*_) *δ* 169.0, 152.4, 142.0, 110.4, 106.7, 35.4, 22.4; **HRMS** (ESI-MS): Calculated for C_7_H_10_NO_2_ [M+H]^+^: 140.07061; found: 140.07070.

#### Preparation of *N*-((4-(cyclopropanecarbonyl)furan-2-yl)methyl)acetamide (13)

AlCl_3_ (2.88 g, 21.57 mmol) and cyclopropanecarbonyl chloride (850 μL, 9.35 mmol) were dissolved in DCM (15 mL) at 0°C and the mixture was stirred at rt for 30 min. Furan **12** (1 g, 7.19 mmol) was added the reaction was kept stirring at rt overnight. H_2_O and EtOAc were added and after extraction the combined organic layers were concentrated *in vacuo*. Silica gel column chromatography (1% → 2% MeOH/DCM) yielded **13** (861 mg, 58%). ^**1**^**H NMR** (500 MHz, DMSO-*d*_*6*_) *δ* 8.44 (s, 1H), 7.51 (d, *J* = 3.5 Hz, 1H), 6.48 (d, *J* = 3.5 Hz, 1H), 4.32 (d, *J* = 5.7 Hz, 2H), 2.64 (tt, *J* = 7.2, 5.4 Hz, 1H), 1.86 (s, 3H), 1.07–0.88 (m, 4H); ^**13**^**C NMR** (126 MHz, DMSO-*d*_*6*_) *δ* 187.4, 169.3, 157.8, 151.4, 119.5, 109.4, 35.7, 22.4, 16.6, 10.4; **HRMS** (ESI-MS): Calculated for C_11_H_14_NO_3_ [M+H]^+^: 208.09682, found: 208.09768.

#### Preparation of *N*-((4-(cyclopropylmethyl)furan-2-yl)methyl)acetamide (14)

A solution of **13** (200 mg, 0.97 mmol) in concentrated HCl (5 mL) and stirred overnight at rt. The reduced crude product (591 mg, 3.58 mmol) was used without any further purification. After dissolving in TFA (11 mL), TES (1.374 mL, 8.6 mmol) was added dropwise and the resulting mixture was stirred at rt overnight. H_2_O and cold saturated NaHCO_3_ were added to the reaction that was subsequently thoroughly extracted with EtOAc. The combined organic layers were concentrated *in vacuo* and silica gel column chromatography (1% → 5% MeOH/DCM) yielded the target compound **14** as a hydrochloride salt (620 mg, 92%). ^**1**^**H NMR** (600 MHz, DMSO-*d*_*6*_) *δ* 8.28 (s, 3H), 6.41 (d, J = 3.1 Hz, 1H), 6.14 (d, J = 3.1 Hz, 1H), 3.66 (t, J = 6.4 Hz, 2H), 3.17 (s, 1H), 2.63 (t, J = 7.3 Hz, 2H), 1.73 (m, 5H); ^**13**^**C NMR** (151 MHz, DMSO-*d*_*6*_) *δ* 156.1, 145.8, 110.9, 106.3, 45.0, 35.2, 31.4, 26.5, 24.8; **HRMS** (ESI-MS): Calculated for C_9_H_15_NOCl [M+H]^+^: 188.08367 and 190.08072; found: 188.08411 and 190.08146.

#### Preparation of 2-(4-fluorophenyl)-4,5-dihydro-1*H*-imidazole (16)

4-Fluorobenzaldehyde **15** (1.28 mL, 12.1 mmol) was dissolved in *t*BuOH (20 mL) and ethane-1,2-diamine (1.1 mL, 15.72 mmol) was added. After 30 min of stirring at rt, K_2_CO_3_ (2.5 g, 18.14 mmol) and I_2_ (9.2 g, 36.28 mL) were added and the resulting mixture was heated to 70°C and stirred for another 3.5 h. The reaction was quenched with saturated Na_2_SO_3_ and extracted with saturated NaHCO_3_ and EtOAc. The combined organic layers were concentrated under reduced pressure and silica gel column chromatography (4% → 10% MeOH/DCM → 6% MeOH/DCM + 1% NH_3(aq)_) yielded **16** (1.1 g, 54%). ^**1**^**H NMR** (500 MHz, DMSO-*d*_*6*_) *δ* 7.92–7.85 (m, 2H), 7.48 (br, 1H), 7.28 (t, J = 8.9 Hz, 2H), 3.63 (s, 2H, 2H); ^**13**^**C NMR** (126 MHz, DMSO-*d*_*6*_) *δ* 163.4 (d, J = 247.7 Hz), 162.7, 129. 6 (d, J = 8.8 Hz, 2C), 126.4 (d, J = 2.8 Hz), 115.2 (d, J = 21.7 Hz, 2C), 49.2; **HRMS** (ESI-MS): Calculated for C_9_H_10_N_2_F [M+H]^+^: 165.08225; found: 165.08209.

#### Preparation of 4-(4-acetylpiperazin-1-yl)benzaldehyde (17a)

4-Fluorobenzaldehyde **15** (619 μL, 5.85 mmol), 1-acetylpiperazine (500 μL, 3.90 mmol) and Na_2_CO_3_ (620 mg, 5.85 mmol) were dissolved in H_2_O (25 mL) and the stirred at 100°C overnight. After extraction with DCM, the combined organic layers were concentrated under reduced pressure and silica gel column chromatography (3% MeOH/DCM) yielded **17a** (942 mg, 58%). ^**1**^**H NMR** (500 MHz, DMSO-*d*_*6*_) *δ* 9.73 (s, 1H), 7.73 (d, J = 8.9 Hz, 2H), 7.04 (d, J = 8.9 Hz, 2H), 3.65–3.54 (m, 4H), 3.50–3.44 (m, 2H), 3.42–3.36 (m, 2H), 2.04 (s, 3H); ^**13**^**C NMR** (126 MHz, DMSO-*d*_*6*_) *δ* 190.2, 168.42, 154.3, 131.4, 126.4, 113.2, 46.3, 46.0, 44.9, 40.3, 21.1; **HRMS** (ESI-MS): Calculated for C_13_H_17_N_2_O_2_ [M+H]^+^: 233.12845; found: 233.12871.

#### Preparation of 4-morpholinobenzaldehyde (17b)

4-Fluorobenzaldehyde **15** (1.8 mL, 17.24 mmol), 1-acetylpiperazine (1 mL, 11.49 mmol) and Na_2_CO_3_ (1.83 g, 17.24 mmol) were dissolved in H_2_O (40 mL) and the stirred at 100°C overnight. After extraction with DCM, the combined organic layers were concentrated under reduced pressure and silica gel column chromatography (20% EtOAc/PE) yielded **17b** (2.36 g, 55%). ^**1**^**H NMR** (400 MHz, DMSO-*d*_*6*_) *δ* 9.97 (s, 1H), 9.73 (s, 1H), 8.11–7.89 (m, 2H), 7.72 (d, J = 8.9 Hz, 2H), 7.43 (q, J = 8.4 Hz, 2H), 7.03 (d, J = 8.9 Hz, 2H), 3.72 (m, 4H), 3.32 (m, 4H); ^**13**^**C NMR** (101 MHz, DMSO-*d*_*6*_) *δ* 191.0, 154.9, 131.7, 126.7, 118.3, 65.8, 46.6; **HRMS** (ESI-MS): Calculated for C_11_H_14_NO2 [M+H]^+^: 192.10191; found: 192.10186.

#### Preparation of 3-(3-(trifluoromethyl)phenyl)-1*H*-pyrazol-5-amine (21)

3-oxo-3-(3-(trifluoromethyl)phenyl)propanenitrile **20** (1.2 g, 5.63 mmol) was dissolved in EtOH (10 mL) and hydrazine (141 μL, 8.80 mmol) was added dropwise. The reaction mixture was stirred for 1 h at rt and then overnight at reflux. After cooling down to rt, addition of H_2_O (twice the reaction volume) led to the precipitation of **21** (630 mg, 50%) as a white solid that was subsequently filtered off and collected. In bigger scales occurring impurities could be removed by an additional work up with NaHCO_3_/10% MeOH/DCM. ^**1**^**H NMR** (500 MHz, DMSO-*d*_*6*_) *δ* 11.89 (d, *J* = 220.0 Hz, 1H), 7.98 (s, *J* = 9.9 Hz, 1H), 7.94 (s, 1H), 7.59 (s, 2H), 5.85 (d, *J* = 67.9 Hz, 1H), 4.88 (d, *J* = 218.2 Hz, 2H).

#### Preparation of 4-(trifluoromethyl)-3-(3-(trifluoromethyl)phenyl)-1,7-dihydro-6*H*-pyrazolo[3,4-*b*]pyridin-6-one (2)

Ethyl 4,4,4-trifluoro-3-oxobutanoate (139 μL, 0.95 mmol) and **21** (180 mg, 0.79 mmol) were dissolved in AcOH (5 mL) and stirred overnight under refluxing conditions. After cooling to rt, ice-water was added to the stirring reaction mixture and led to precipitation of crude product that was subsequently filtered off. Silica gel column chromatography (60% → 100% EtOAc/PE) and washing of the concentrated collected fractions with hexane yielded pure **2** (145 mg, 53%). ^**1**^**H NMR** (500 MHz, DMSO-*d*_*6*_) *δ* 13.82 (s, 1H), 12.41 (s, 1H), 7.88 (s, 1H), 7.84–7.77 (m, 2H), 7.76–7.69 (m, *J* = 7.2 Hz, 1H), 6.61 (s, 1H); **HRMS** (ESI-MS): Calculated for C_14_H_8_N_3_OF_6_ [M+H]^+^: 348.05661; found: 348.05715.

#### Preparation of 4-cyclopropyl-3-(3-(trifluoromethyl)phenyl)-1,7-dihydro-6*H*-pyrazolo[3,4-*b*]pyridin-6-one (22)

Ethyl 3-cyclopropyl-3-oxopropanoate (131 μL, 0.85 mmol) and **21** (160 mg, 0.70 mmol) were dissolved in AcOH (5 mL) and stirred overnight under refluxing conditions. After cooling to rt, ice-water was added to the stirring reaction mixture and led to precipitation of crude product that was subsequently filtered off and washed with hexane to yield **22** (113 mg, 51%). ^**1**^**H NMR** (500 MHz, DMSO-*d*_*6*_) *δ* 12.36 (s, 1H), 8.28 (d, *J* = 5.3 Hz, 2H), 7.77 (d, *J* = 7.8 Hz, 1H), 7.71 (t, *J* = 8.0 Hz, 1H), 6.71 (s, 1H), 5.46 (s, 1H), 1.96–1.91 (m, 1H), 1.14–1.06 (m, 2H), 1.03–0.96 (m, 2H); ^**13**^**C NMR** (126 MHz, DMSO-*d*_*6*_) *δ* 156.3, 156.1, 151.2, 142.8, 133.6, 130.1, 129.9, 129.5, 125.2, 122.2, 90.5, 86.0, 13.0, 9.2; **HRMS** (ESI-MS): Calculated for C_16_H_13_N_3_OF_3_ [M+H]^+^: 320.10052; found: 320.10100.

#### Preparation of 4-methyl-3-(3-(trifluoromethyl)phenyl)-1,7-dihydro-6*H*-pyrazolo[3,4-*b*]pyridin-6-one (23)

Ethyl 3-oxobutanoate (110 μL, 0.85 mmol) and **21** (160 mg, 0.70 mmol) were dissolved in AcOH (5 mL) and stirred overnight under refluxing conditions. After cooling to rt, ice-water was added to the stirring reaction mixture and led to precipitation of crude product that was subsequently filtered off and washed with hexane to yield **23** (123 mg, 60%). ^**1**^**H NMR** (500 MHz, DMSO-*d*_*6*_) *δ* 12.44 (s, 1H), 8.29 (s, 2H), 7.80–7.74 (m, 1H), 7.74–7.68 (m, 1H), 6.75 (s, 1H), 5.63 (s, 1H), 2.32 (s, 3H); ^**13**^**C NMR** (126 MHz, DMSO-*d*_*6*_) *δ* 156.0, 151.3, 150.5, 142.9, 133.6, 130.1, 129.9, 125.3, 122.2, 95.4, 85.9, 18.6; **HRMS** (ESI-MS): Calculated for C_14_H_10_N_3_OF_3_Na [M+Na]^+^: 316.06682; found: 316.06720.

### Protein expression, purification and crystallization

The expression and purification of inactive, non-phosphorylated p38*α* wt MAPK was done as previously reported [20]. Briefly, an N-terminal His_6_-p38*α* wt construct was transformed in to *E*. *coli* BL21(DE3) and expressed overnight at 18°C. The protein was purified by Ni^2+^-NTA-affinity chromatography, followed by anion exchange and size exclusion chromatography after removal of the His-tag by proteolytic cleavage. For SPR experiments the corresponding protein batch did not undergo His-tag cleavage. The pure protein was subsequently concentrated to 10–30 mg/mL, aliquoted, flash frozen in liquid N_2_ and stored at -80°C.

**Fig 2:**
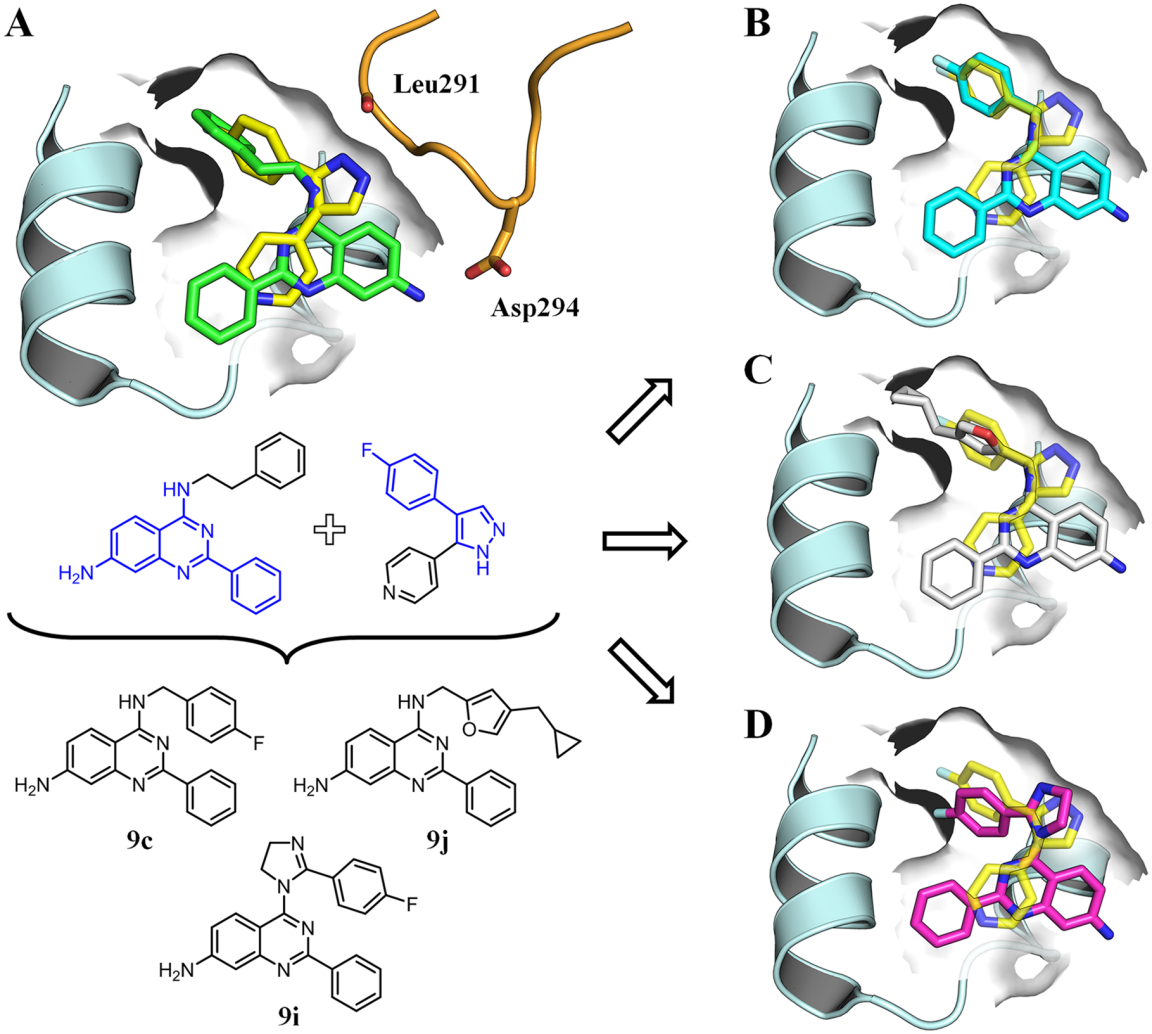
Compound design. (A) Design of LiPoLis based on the alignment of the crystal structures of **3** (green) and **1** (yellow) in complex with p38*α* (PDB-codes: 4DLJ and 3HVC). Overlay of **1** and modeled structures of (B) **9c** (cyan), (C) **9j** (white) and (D) **9i** (magenta) showcasing the proposed binding modes.

**Fig 3:**
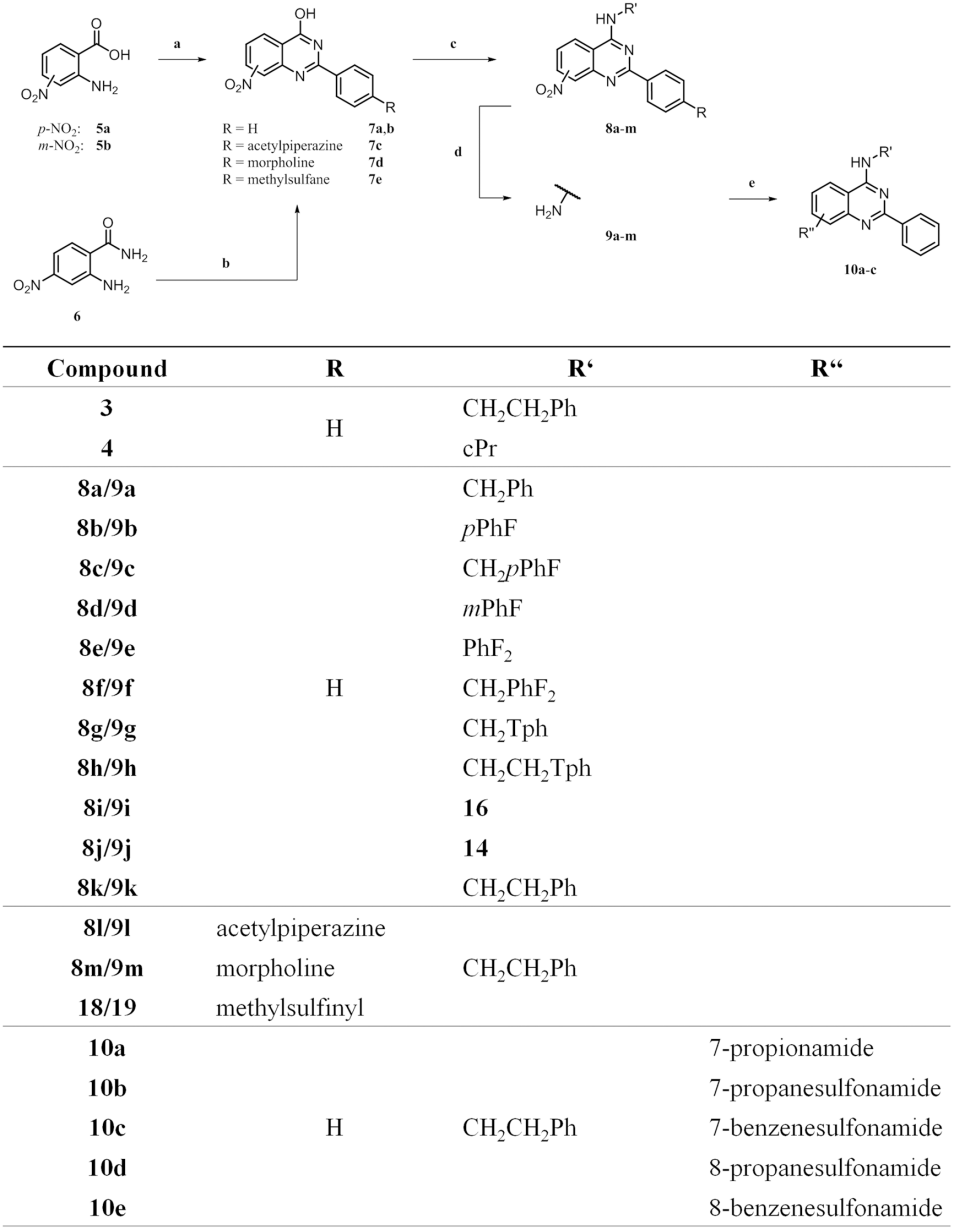
^*a*^ Synthesis and identity of 2-arylquinazolines. ^*a*^ Reagents and conditions: (a) method A: benzamidine hydrochloride hydrate, AcOH, 2-methoxyethanol, 130°C, 18 h, 18–32%; method B: benzoic anhydride, formamide, 200°C, 5 min, MW, 31–37%; (b) method C: aldehyde, NaHSO_3_, *p*TSA, DMAc, 155°C, 18 h, 10–34%; (c) method A: 1) SOCl_2_, DMF, 80°C, 4 h, 2) amine, DIPEA, DCM/*i*PrOH (3:2), rt, 18 h, 66–95%; method B: 1) HCCP, DIPEA, MeCN, rt, 1 h, 2) amine, rt, 18 h, 68–87%; (d) method A: 10% Pd/C, ammonium formate, EtOH, 80°C, 1–3 h, 56–96%; method B: Fe, NH_4_Cl, MeOH:H_2_O (4:1), 80°C, 3–6 h, 81–87%; (e) acyl chloride, DIPEA, DCM, 0°C to rt or 50°C, 1–6 h, 69–80%.

**Fig 4:**
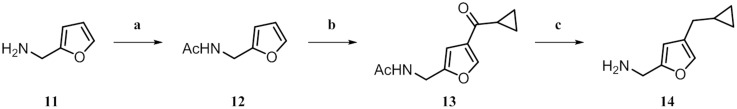
^*a*^ Synthesis of amine building block 14. ^*a*^ Reagents and conditions: (a) Ac_2_O, DCM, rt, 18 h, 88%; (b) AlCl_3_, cyclopropanecarbonyl chloride, DCM, rt, 30 min, 58%; (c) 1) HCl (conc.), rt, 18 h, 2) TFA, TES, rt, 18 h, 92%.

Various LiPoLis were co-crystallized with p38*α* wt using conditions similar to those as described previously [14]. Briefly, protein-ligand complexes were prepared by mixing 40 μL p38*α* wt (10 mg/mL) with 1 μL compound (50 mM in DMSO) and incubated for 60 min on ice. The samples were centrifuged at 13,000 rpm for 10 min to remove excess ligand. Crystals were grown in 24-well crystallization plates (EasyXtal Tool, Quagen, Hilden, Germany) using the hanging drop vapor diffusion method and by mixing 1.5 μL protein-ligand solution with 0.5 μL reservoir (100 mM MES pH 5.6–6.2, 20–30% PEG4000 and 50 mM BOG). In some cases, crystals were obtained when BOG was absent in the reservoir solution and when 125 μM BIRB-769 were used instead of BOG, respectively. The crystals were protected using 25% PEG400 before they were flash frozen in liquid N_2_. Diffraction data of the p38*α*-ligand complexes were collected at the PX10SA beam line of the Swiss Light Source (PSI, Villigen, Switzerland) using wavelengths close to 1 Å. The datasets were integrated with XDS [21] and scaled using XSCALE [21]. The complex structures were solved by molecular replacement with PHASER [22] using the published p38*α* structure of **4** (PDB: 4DLI) [13] as template. Molecules in the asymmetric unit were manually modified using the program COOT [23]. Inhibitor topology files were generated using the Dundee PRODRG server [24]. Final refinement was done employing the PDB-redo server [25] and PHENIX [26]. Refined structures were validated by Ramachandran plot analysis with RAMPAGE [27]. Data collection, structure refinement statistics, PDB-entry codes and the Ramachandran plot results are shown in S2 Table. Electron densities used for the omit maps were generated *via* a simulated annealing refinement with ligand and water molecule occupancy set to”0”. PyMOL [28] was used to produce Figs 1–4 and S5 Fig.

### Surface plasmon resonance

For the kinetic measurements a SPR-2/4 from Sierra Sensors (Hamburg, Germany) was used. All experiments were performed with a constant flow rate of 25 μL/min of running buffer (1x PBS, 150 mM NaCl, pH 7.4, 0.001% Triton^™^ X-100, 3% DMSO). At first, a trisNTA sensor was made by standard amine coupling of amino-trisNTA [29] (100 mM) to a commercially available HCA sensor (Sierra Sensors) using a 1:1-cocktail of EDC/NHS (0.4 M/50 mM) for activation and ethanol amine (1 M, pH 8.5) for blocking of unreacted surfaces (reactions outside the device; no flow). Then, the sensor was mounted to the SPR-2/4 and 50 μL of 200 μM Ni(II) acetate were injected over a reference and an active spot on the sensor surface, followed by subsequent injection of 50 μL inactive His_6_-p38*α* wt (2 mg/mL in running buffer) over the active spot and 50 μL His_6_-peptide (MBL) (2 mg/mL in running buffer) over the reference spot. After 30 min of baseline stabilisation due to dissociation of the His-tagged kinase from the trisNTA surface an immobilization level of ca. 3000–4000 RU was reached, with a remaining, negligible drift of ca. 2 RU/min.

A concentration series of LiPoLis and SB203580 (200 nM—6 μM, 1–30 μM and 5–500 nM, respectively) along with blank injections for referencing, were injected over 2 and 5 min, respectively, to both, the reference and the active spot (association), followed by washing with running buffer for 1 min and 4 min, respectively (dissociation). No surface regeneration was needed after compound injection. For solvent calibration 15 μL injections of 4.4–5.6% DMSO in running buffer were run. Finally, multiple injections of 100 μL 0.5 M EDTA regenerated the trisNTA sensor completely. Raw data was processed und globally fitted with Analyser R2 v0.2.3.8 (Sierra Sensors).

### Thermal shift assay

Melting curves of p38α wt were measured at two compound concentrations (50, 10 μM). Compounds in 10 mM DMSO stock solutions were diluted to 2.5 mM and 0.5 mM with DMSO, respectively. From each of the dilution 0.8 μL were added to a well of a 96-well plate (TW-MT, Biozym), except water and reference (DMSO) wells. A protein stock solution (30 mg/mL p38α wt) was diluted to 1 μM in sample buffer (10 mM HEPES, 150 mM NaCl, 5x dye, pH 7.5), already containing the dye SYPRO^®^ orange (1000x diluted 5000x stock in DMSO, Sigma) and kept protected from light. To each well of the measurement plate were added 39 μL of this protein solution and the samples were appropriately mixed. After the plates were sealed with optical foil and spun down (200 g, rt, 1 min), they were subsequently placed in a LightCycler^®^ 480 II (Roche). Experiment was carried out at 492 nm excitation and 610 nm emission wavelength and the temperature ramping from 25°C to 95°C with a rate of 1°C/min. ΔT was determined from the difference to DMSO control samples.

## Results and discussion

### Structure-based design and synthesis of a focused 4-amino-2-arylquinazoline compound library

Starting from the crystal structures of **3** and **4** in complex with p38*α* (PDB-codes: 4DLI and 4DLJ) and some knowledge of the underlying binding mode, we employed a rational approach for the design and synthesis of new 4-amino-2-arylquinazolines. As a major design aspect, we made no changes to the main interactions contributing to ligand binding, namely the parallel-displaced *π*-*π* stacking between the quinazoline core and the aromatic side chain of Trp197 and a direct hydrogen bond between the anilinic amine and the Asp294 carboxylic acid function. The deep sub-pocket decorated with lipophilic amino acid residues is occupied by a hydrophobic moiety, in this case a phenethyl substituent in the 4-position. This site was thought to be suitable for derivatization to potentially increase compound affinity towards the kinase, since it has been shown that small molecules with differently sized moieties at this position could bind to the LP, with **3** being the most demanding LiPoLi observed thus far [13]. Since previous studies showed that **3** was poorly soluble in aqueous media, we selected the 2-phenyl ring pointing outside the LP towards the solvent for modification with solubilizing groups (Fig 1B). Furthermore, the 7-position of the scaffold should be easily derivatized while maintaining the NH-group as a hydrogen bond donor. Taken together, these observations led us to choose the lipophilic moiety in the 4-position, the solvent-exposed 2-phenyl group as well as the 7-amine as putative sites of modification for the preparation of a diverse quinazoline-based compound library (Fig 1C).

The alignment of LiPoLis **1** and **3** (PDB-codes: 3HVC and 4DLJ) followed by structural analysis prompted us to our initial concepts of derivatization of the quinazoline core scaffold (Fig 2A). In our first design approach, we increased the size of the lipophilic group in the 4-position by introducing fluoro- and difluorophenyl substituents (**9b**-**f**) (Fig 2B). These hydrophobic elements might fill out the sub-pocket and thereby form additional favorable interactions, *e*.*g*., halogen bonds to backbone carbonyls [30]. Furthermore, the binding of fluorinated compounds to receptors may be entropically favored due to the liberation of water by desolvation, particularly at hydrophobic moieties [31]. Substitution of the phenyl ring with a thiophene (Tph) moiety and variation of the linker length to the scaffold (**9g**,**h**) emerged as interesting alternatives since thiophene is a bioisostere with respect to the phenyl group and features different chemical and electronic properties [32]. Using a shorter methylene linker may provide sufficient space for expansion of the five-membered ring, which was the basis of the design of **9j** (Fig 2C). Aliphatic moieties like the present cyclopropyl group in building block **14** that was used for the synthesis of **9j** can make a greater contribution to the hydrophobic interactions than aromatic substituents, and thus these aliphatic structures may be advantageous for improving ligand affinity [33]. As an alternative strategy, we also designed a hybrid compound (**9i**), which retained the scaffold of **3** while the 4-position was derivatized with a fragment of the known LiPoLi **1** (Fig 2D).

Furthermore, the solvent exposed 2-phenyl ring and the amine in the 7-position were chosen as derivatization sites for the introduction of solubilizing groups, such as acetylpiperazine (**9l**), morpholine (**9m**), methylsulfinyl (**19**), amides and sulfonamides (**10a-e**). Also, 8-amino derivative **9k** was designed to investigate any impact on the binding affinity, since new hydrogen bonds to the amino acid chains could be formed. The structural basis for this assumption is the relatively high density of polar amino acid side chains in this region of the LP, such as Asp294 being directly involved in the binding of **3**.

We developed a common synthetic route which successfully led to the proposed target compounds (Fig 3). The quinazoline cores **7a**-**d** were built up in the reaction by use of anthranilic acids **5a** and **5b** with benzamide and benzoic anhydride moieties, respectively [34, 35], or by using anthranilic amide **6** and aldehyde building blocks [36, 37]. Substitution in the 4-position with an amine took place after activation of the hydroxyl group with hexachlorocyclotriphosphazene (HCCP) [38], yielding the nitro compound precursors **8a**-**n**, which were subsequently reduced to the corresponding amines **9a**-**m**. These were feasible substrates to undergo nucleophilic substitution with either carboxylic acids or sulfonyl chlorides to yield compounds **10a**-**e**.

**Fig 5:**
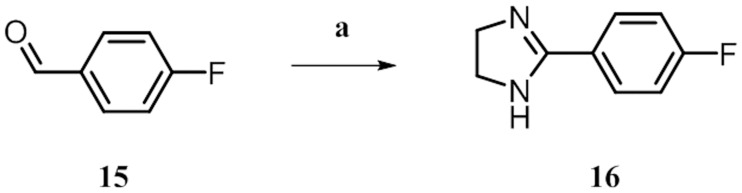
^*a*^ Synthesis of amine building block 16. ^*a*^ Reagents and conditions: (a) ethane-1,2-diamine, K_2_CO_3_, I_2_, *t*BuOH, 70°C, 3.5 h, 54%.

Some LiPoLis required the synthesis of building blocks to be used for coupling to the scaffold (**14**, **16**) or as components for condensation to the quinazoline core (**17a**,**b**). Starting with the protection of **11** *via* acetylation and subsequent Friedel-Crafts acylation of the furan ring **12**, the final amine **14** was generated by reduction and simultaneous deprotection of intermediate compound **13** under acidic conditions (Fig 4). Dihydroimidazole **16** was prepared by condensation of 4-fluorobenzaldehyde **15** with ethylene diamine (Fig 5).

**Fig 6:**
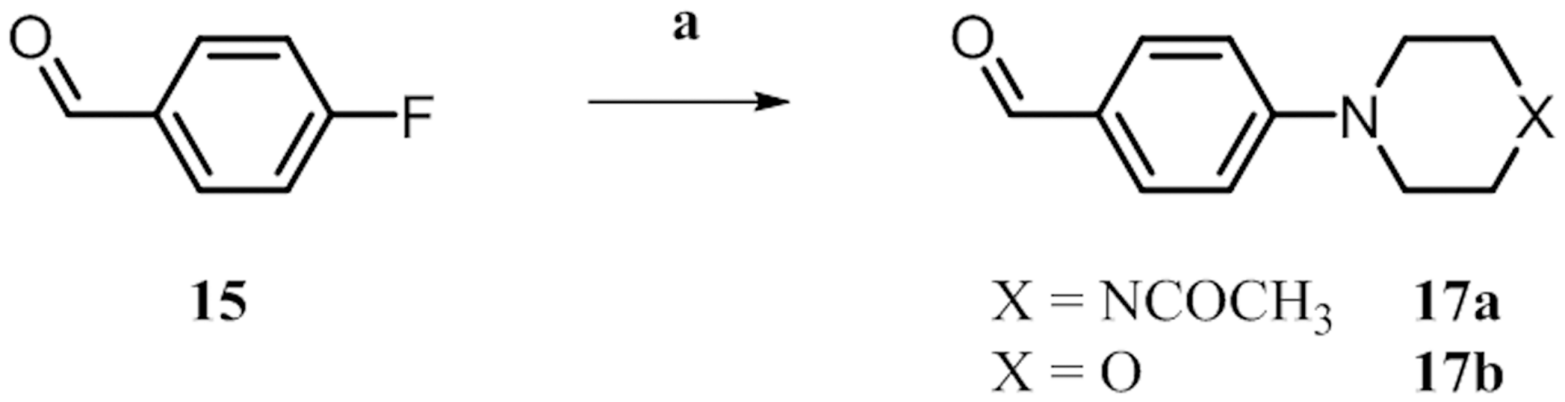
^*a*^ Synthesis of aldehyde building blocks 17a and 17b. ^*a*^ Reagents and conditions: (a) amine, Na_2_CO_3_, H_2_O, reflux, 18 h, 55–58%.

**Fig 7:**
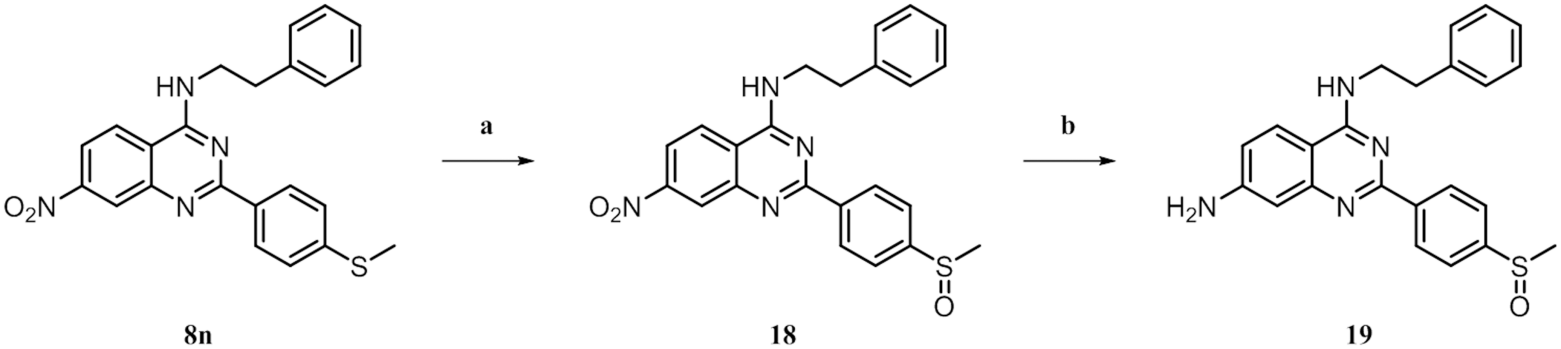
^*a*^ Synthesis of 19. ^*a*^ Reagents and conditions: (a) *m*CPBA, DCM, rt, 3 h, 87%.

To decorate the quinazolines **9l**,**m** with corresponding solubilizing groups, the aldehydes **17a** and **17b** were synthesized by substituting 4-fluorobenzaldehyde **15** with acetylpiperazine and morpholine, respectively (Fig 6). For the generation of **19** starting from **7a**, the intermediate **8n** was oxidized to the sulfinyl compound **18** that was subsequently reduced under mild conditions to give the final amine (Fig 7). Following these procedures, we synthesized a library of over 30 compounds.

**Fig 8:**
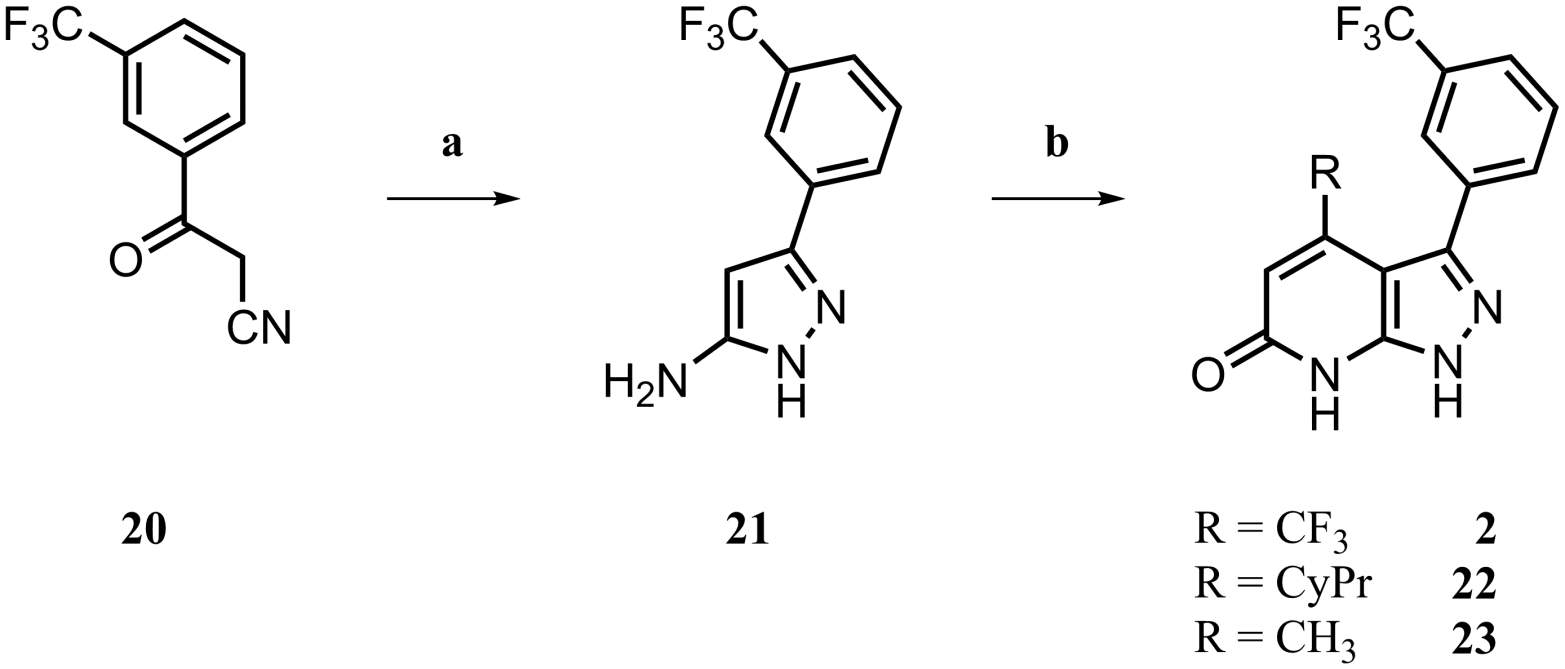
^*a*^ Synthesis of 2, 22 and 23. ^*a*^ Reagents and conditions: (a) hydrazine, EtOH, reflux, 21 h, 50%; (b) oxobutanoate, AcOH reflux, 19 h, 51–60%.

As a potential control ligand, we also synthesized **2** [17], starting with the condensation of nitrile component **20** and hydrazine [39]. The resulting pyrazolamine **21** was converted with 4,4,4-trifluoro-3-oxobutanoate to yield the final product **2**. Analogues **22** and **23** were designed to explore possible chemical space with a putative alternative LiPoLi scaffold and were easily generated by using the corresponding oxobutanoates (Fig 8). To subsequently validate our design concept and to confirm that the newly developed LiPoLis would address the LP of the p38*α* MAPK, we undertook SPR experiments.

### Surface plasmon resonance

For SPR measurements, the His_6_-p38*α* wt was immobilized on a trisNTA [29] sensor. The system was tested with the active site inhibitor SB203580 that showed reproducible results (k_on_ = 1.43 · 10^6^ (M · s)^-1^, k_off_ = 2.43 · 10^−3^ s^-1^, *K*_D_ = 1.7 nM) in good agreement with the literature [34, 40] (S1A Fig). Thus, the chosen assay was subsequently used for the characterisation of the synthesized library of quinazoline-based LiPoLis.

Injection of LiPoLi samples gave a concentration dependent response for some of the synthesized derivatives, indicating a specific binding event to the p38*α* kinase domain (S1B–S1D Fig) since other LiPoLis did not show any increasing signal upon compound injection (S1F Fig). Hence, this renders the separation between actual ligands and non-binding molecules to the immobilized protein and the identification of tolerated structural modifications to the LiPoLis by SPR possible. Concerning the ligands that didn’t show any response, all nitro derivatives **8a-m** as well as 7- and 8-substituted LiPoLis **10a-e** can be considered not to bind to p38*α* under the chosen experimental conditions. Notably, also reference compound **2** and its derivatives **22** and **23** did not show any response in the SPR experiments with increasing analyte concentrations.

Those ligands that were identified to positively bind to p38*α* MAPK in these studies all exhibited a similar shape of the detected sensorgram as the initial lead compound **3**. Starting at baseline level, sensorgrams showed an increasing response during analyte injection in a concentration-dependent manner, which indicates a positive binding event to the immobilized protein. Unspecific binding to the sensor surface of the reference channel could be essentially excluded, since corresponding sensorgrams did not represent any signal indicating unwanted interactions with the reference surface blocked with the His_6_-peptide. At higher concentrations the tested analytes did steadily bind to the active sensor surface without reaching an equilibrium, indicating accumulating effects to the kinase. This behaviour complicates the determination of kinetic parameters in terms of association as a 1:1-Langmuir fit model did not properly represent the binding event. However, the observed effect was reversible, as baseline level was recovered after switching from compound injection to running buffer flow.

Compounds that showed a characteristic response in the SPR measurements allow conclusions regarding the tolerated chemical space concerning compound modifications that can be made to the 2-arylquinazoline scaffold without impairing the ability to address the LP. The exemplified results for the characterisation of **3**, **9c** and **9m** showcase the commonly observed shape of sensorgrams of the tested LiPoLis, that were reflected by slow association and significantly fast dissociation (k_off_ = 0.04–0.06 s^-1^) (S1B–S1D Fig), typically leading to weak binding affinities. Substituting the phenethyl moiety in 4-position with bioisosteres in form of thiophenes **9g** and **9h** or replacement by fluorophenyl residues (**9b**-**d**) gave comparable and reproducible sensorgrams. On note, derivatives **9e**,**f** carrying the difluorophenyl moiety appeared to lose any affinity to the protein compared to the other 4-aminoquinazolines. In direct comparison to **3**, the solubilizing group-bearing derivatives **9l**,**m** showed a significantly faster association before reaching the equilibrium state at lower ligand concentrations (S1D Fig). Thus, these results demonstrate, that even sterically more demanding moieties are tolerated at the phenyl ring in the 2-postion. In one case, the binding signal of **9j** detected from the active channel was apparently masked by non-specific binding occurring at both, the active surface and the blocked reference surface, reflected in pseudo-irreversible binding after double-referencing (S1E Fig).

In summary, we could establish a robust SPR assay system that was set up using the known inhibitor SB203580 and generated reliable and reproducible data for the characterisation of the presented ligands. Weak affinity of the analytes towards the target kinase was generally observed in SPR and orthogonal assays, *e*.*g*., thermal shift assays (S3 Fig). To further characterise the designed LiPolis regarding the exact binding mode when bound to p38*α* MAPK co-crystallization experiments were conducted.

### Crystallography

For the crystallization, we pursued different strategies that resulted in different co-crystallized structures of p38*α* MAPK in complex with LiPoLi quinazolines. Crystal growth varied depending on the experimental protocol followed and compounds used. When previously published conditions were used in the presence of BOG [14], crystals and the corresponding protein structures were usually obtained that accommodated the detergent in the LP. BOG is known to bind to the LP and the concentration used under these conditions may compete with the LiPoLi quinazolines for the occupation of the LP. Therefore, we also conducted crystallization trials in the absence of BOG. A third crystallization method focused on using the active site inhibitor BIRB-769 to stabilize the kinase and thereby facilitate crystal growth and eventually binding of the LiPoLis. Generally, crystal growth took place spontaneously or within 21 days, yielding needle-shaped crystals when BOG or BIRB-796 were present. More spherical-shaped crystals were obtained when the protein was exclusively incubated with the LiPoLis (S4 Fig). Protein crystals showing the spherical morphology usually were occupied with bound LiPoLis to the LP and could thereby serve as an indicator for successful co-crystallization (a summary of these findings can be found in S1 Table).

When crystallization trials were set up with ligands bearing an alternative substituent other than an amine in the 7-position, immediate precipitation of the protein was observed. Thus, no crystals could be obtained for nitro compounds **8a**-**n** and the derivatives **9k**, **10a**-**e** as well as **2** and its analogues. This outcome indicated that sterically demanding substituents in the 7-position might interfere with protein binding, resulting in the loss of the previously observed H bond between the amine and Asp294.

**Fig 9:**
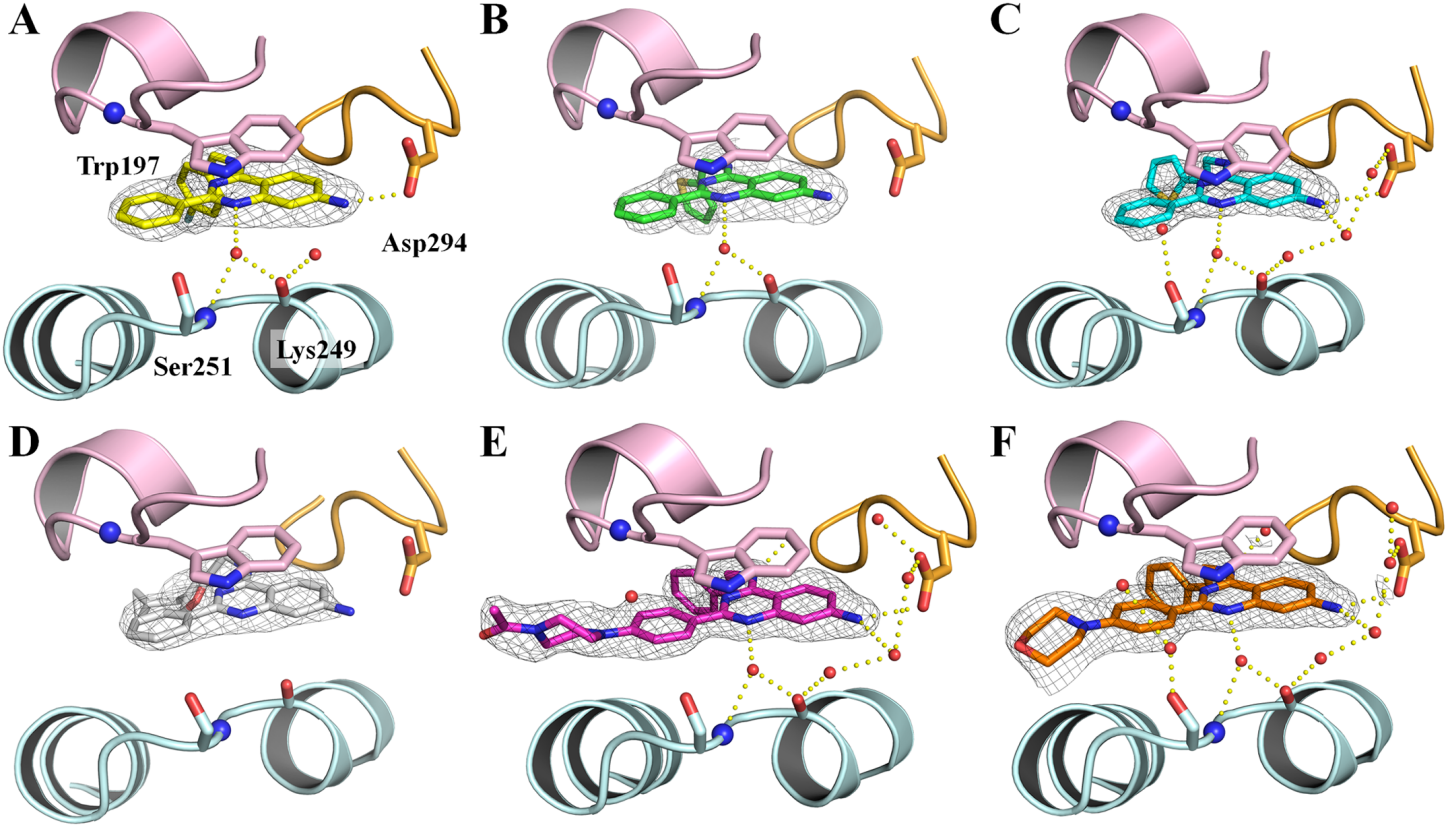
Crystal structures of LiPoLis in complex with p38*α*. Diagrams of the experimental electron densities of (A) **9c** (yellow), (B) **9g** (green), (C) **9h** (cyan), (D) **9j** (white), (E) **9l** (magenta) and (F) **9m** (orange). At resolutions ranging from 1.85 to 2.4 Å; 2mFo-DFc map (grey) contoured at 1.0*σ*. Water molecules are shown as red spheres. Hydrogen-bond interactions of the ligands with the protein and water molecules are illustrated by yellow dotted lines. All LiPoLis bind to the LP flanked by helices 1L14, 2L14 and the *α*EF/*α*F loop.

We started our trials of the 4-amino derivatives following the direct co-crystallization protocol and the complex of **9c** was one of the first structures that was successfully solved (Fig 9A). The generally proposed binding mode and key interactions were still maintained, including the phenethyl moiety binding deeply into the lipophilic sub-pocket, the *π*-*π*-interaction between Trp197 and the core quinazoline scaffold, and a direct hydrogen bond between the amine in the 7-position and Asp294 as well as a hydrogen bonding network with surrounding water molecules and amino acid side chains and the protein backbone, *e*.*g*., including Ser251 and Lys249, and partly stabilized by contacts involving crystal symmetry mates, particularly Glu336 (S5 Fig). Furthermore, we obtained crystals for **9g** and **9h** that were conceived as bioisosteres of **3** by substituting the 4-residue with the corresponding thiophene moieties (Fig 9B and 9C). Thus, we demonstrated that the hydrophobic sub-pocket of the LP was capable of harboring that five-membered heterocycle while maintaining the general binding mode described above. These findings were the starting point for the design of **9j** as an “extended derivative" of **9g**, which was achieved by adding another methylene cyclopropyl to the furan ring. We were gratified to observe that the co-crystallization of this LiPoLi succeeded in presence of BIRB-796. It is noteworthy that the binding mode of **9j** within the LP was not significantly different from the previously mentioned key interactions (Fig 9D). The cyclopropyl moiety of **9j** was able to access the deeply buried regions of the LP. Furthermore, the cyclopropyl group did not lead to any perturbation of the protein structure based on the arrangement of adjacent amino acid side chains, as compared to the other LiPoLi bound structures. The difference electron density map was not defined at the position where the cyclopropyl substituent was situated, indicating a significant flexibility and the ability to twist within the lipophilic sub-pocket of the LP.

**Fig 10:**
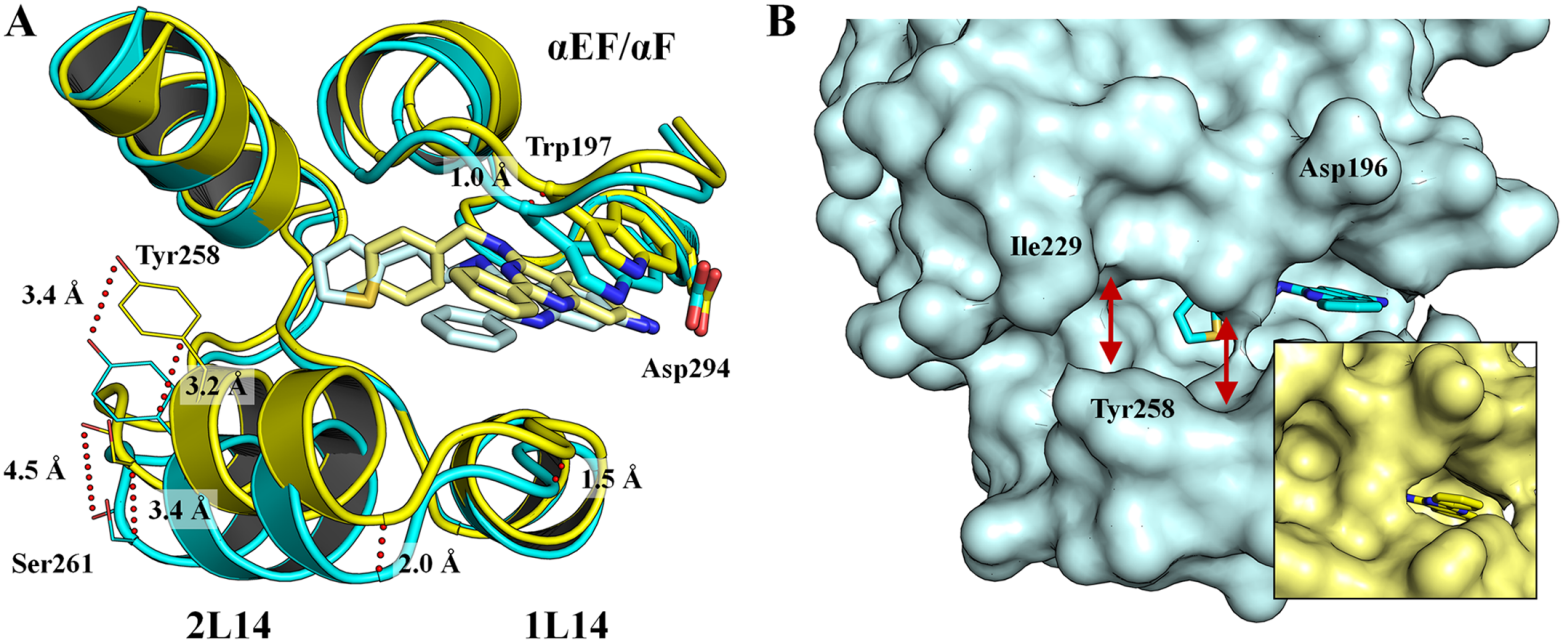
Conformational changes of the LP upon LiPoLi binding. (A) Alignment of the p38*α*-**9c** (cyan) and p38*α*-**9h** (yellow) complex crystal structures. Displacement of helix 2L14 and minor rearrangement of loop *α*EF/*α*F and helix 1L14 (trajectories are shown as red dots); (B) Opening of the LP (red arrows) when **9c** is bound compared to the closed LP in presence of **9h**.

Interestingly, some compounds only crystallized in the absence of BOG in the reservoir solution, and these samples also exhibited a new crystal morphology as observed for **9l** and **9m** (S4 Fig). The co-crystal structures for both compounds were successfully solved and confirmed our design approach as they revealed the same binding geometry as the initial lead compound **3** (Fig 9E and 9F). Compounds **9l** and particularly **9m** were designed to improve the compound’s solubility by introducing a solubilizing group at the phenyl ring in the *para*-position. The 2-phenyl substituent was orientated towards the solvent, similar to **3**, although no interactions with the surrounding water molecules were found. Notably, the network of water molecules surrounding the LP was best resolved in the p38*α*-**9m** complex crystal structure (Fig 9F). Performing a simulated annealing refinement, mFo-DFc omit maps were generated for all co-crystallized LiPoLis and show defined electron density that could be clearly assigned to the corresponding ligands and demonstrated a consistent mode of binding (S6 Fig). Only for **9j**, no defined density of the cyclopropyl moiety within the LP was observed, likely due to conformational flexibility, and **9h** only showed partial density of the quinazoline scaffold, potentially caused by lower occupancy at the chosen experimental conditions. When we compared our new complex crystal structures with p38*α*-**1** and p38*α*-**3**, we commonly observed subtle conformational changes of secondary structure elements shaping the LP. Interestingly, a more significant displacement of helix 2L14 was found in the p38*α*-**9h** complex structure, resulting in an almost complete opening of the lower section of the LP (Fig 10). This underlines a pronounced flexibility of the entire binding site rendering it putatively addressable by even more complex synthetic ligands or yet to be identified biological binding partners.

## Conclusions

Due to their central role in cell signaling pathways, protein kinases are prominent targets in drug research and development. Several diseases are caused by direct dysregulation of the corresponding kinase or their mediators and interaction partners, implying that not only the integrity of enzymatic mechanisms, but also of non-catalytic functions, often referred to as scaffolding functions [10], are mandatory to preserve the sensitive and well-regulated processes within the cell. The successful targeting of scaffolding functions requires detailed knowledge of the participating interaction partners as well as the mechanisms of communication, which include the important structural elements such as binding epitopes and their ligands as well as their biological roles. Here, we set out to target the previously identified LP in p38*α* MAPK. By applying structure-guided derivatization of **3**, we introduced structural variations in the hydrophobic moiety in the 4-position and the functionalization of the 2-phenyl ring and the 7-/8-positions that led to a focused library of over 30 compounds. An SPR assay system was set up to get insight into the kinetics of LiPoLi binding towards p38*α* MAPK. The interactions of the tested ligands and the immobilized kinase was typically described by a slow association phase and saturation followed by fast dissociation. Most of the LiPoLis that showed a characteristic response in the SPR experiments were also successfully co-crystallized with p38*α* MAPK. A series of six complex crystal structures showed that the designed LiPoLis indeed target the LP of p38*α* MAPK and validated our design approaches. The complex structure of **9j** could be solved although in the SPR experiments only unspecific binding to the sensor surface was detected.

In summary, we identified substitution patterns of the LiPoLi scaffold to be crucial for the opening of the LP underlining the flexible nature of this binding site. The characterization of the ligand-binding event by SPR indicated, however, that these LiPoLis are most likely not suitable to serve as functional probes given their weak binding affinities. Anyhow, the results presented here will encourage further compound modifications to focus particularly on the 2-phenyl ring and alternative substitutions to generate more potent LiPoLis to finally dissect the functional role of the lipid pocket in p38*α*.

## Supporting information

S1 Fig Representative sensorgrams of SB20350 and LiPoLis of p38*α* MAPK. Time-dependent changes in resonance units (RU) were detected during the injection of (A) SB203580, (B) **3**, (C) **9h**, (D) **9l**, (E) **9j** and (F) **2** at various concentrations to a sensor surface carrying immobilized His_6_-p38*α*. Global 1:1-Langmuir binding fits are shown as black lines.
(TIF)

S2 Fig Representative sensorgrams of all LiPoLi amine derivatives. Time-dependent changes in resonance units (RU) were detected during the injection of LiPoLis in concentrations ranging from 1 nM—30 μM to a sensor surface carrying immobilized His_6_-p38*α*. Global 1:1-Langmuir and multi-phasic binding fits, respectively, are shown as black lines. LiPoLi nitro derivatives showed no response and are therefore not shown.
(TIF)

S3 Fig Thermal shift assay. A) p38*α* MAPK melting curves and their first derivatives in presence of active site inhibitors and a selection of LiPoLis. Thermal shifts ΔT (°C) were calculated from substraction of melting point in presence of DMSO from measured melting points T_m_ (°C) in presence of compound. B) p38*α* MAPK melting curves and their first derivatives for all presented LiPoLis.
(TIF)

S4 Fig Protein crystals of p38*α* MAPK. Crystals grown in presence of **A) 9g** (needles), **B) 9l** and **C) 9m** (cubic). Crystals were grown at 20°C using 100 mM MES pH 5.6–6.2, 20–30% PEG4000 and 50 mM BOG as reservoir solution.
(TIF)

S5 Fig Water-mediated contact to crystal symmetry mate Glu336. Exemplified for p38*α* in complex with **9c**; symmetry mate is shown in grey, numbers represent H bond distances in Å (PDB: 5N63).
(TIF)

S6 Fig Omit maps of the crystallized LiPoLis. Performing a simulated annealing refinement, mFo-DFc omit maps (green, contoured at 2.5*σ*) were calculated for A) **9c**, B) **9g**, D) **9h**, E) **9j**, E) **9l** and F) **9m**, as well as for surrounding water molecules. 2Fo-Fc maps for the ligands, waters and key residues Trp197 and Asp294 were contoured at 1.0*σ* (blue). Maps indicate partial occupancy for **9h** due to multiple molecules bound to the protein and conformational flexibility of the cyclopropyl moiety in **9j**.
(TIF)

S1 Table Crystallographic statistics of p38α MAPK in complex with LiPoLi derivatives. Statistics for co-crystals with LiPoLis **9c**, **9g**, **9h**, **9j**, **9l** and **9m** (PDBs: 5N63, 5N64, 5N65, 5N66, 5N67 and 5N68). Values in parenthesis refer to the highest resolution shell.
(TIF)

S1 File NMR spectra. (DOCX)

S2 File Overview of collected data regarding LiPoLi characterization. (DOCX)

S3 File Molecular formula strings. (CSV)
