# Supplementary figures and images for: Structure-based design, synthesis and crystallization of 2-arylquinazolines as lipid pocket ligands of p38α MAPK
Source: PLoS One. 2017 Sep 11;12(9):e0184627. doi: 10.1371/journal.pone.0184627 (PMC5593189; doi:10.1371/journal.pone.0184627)

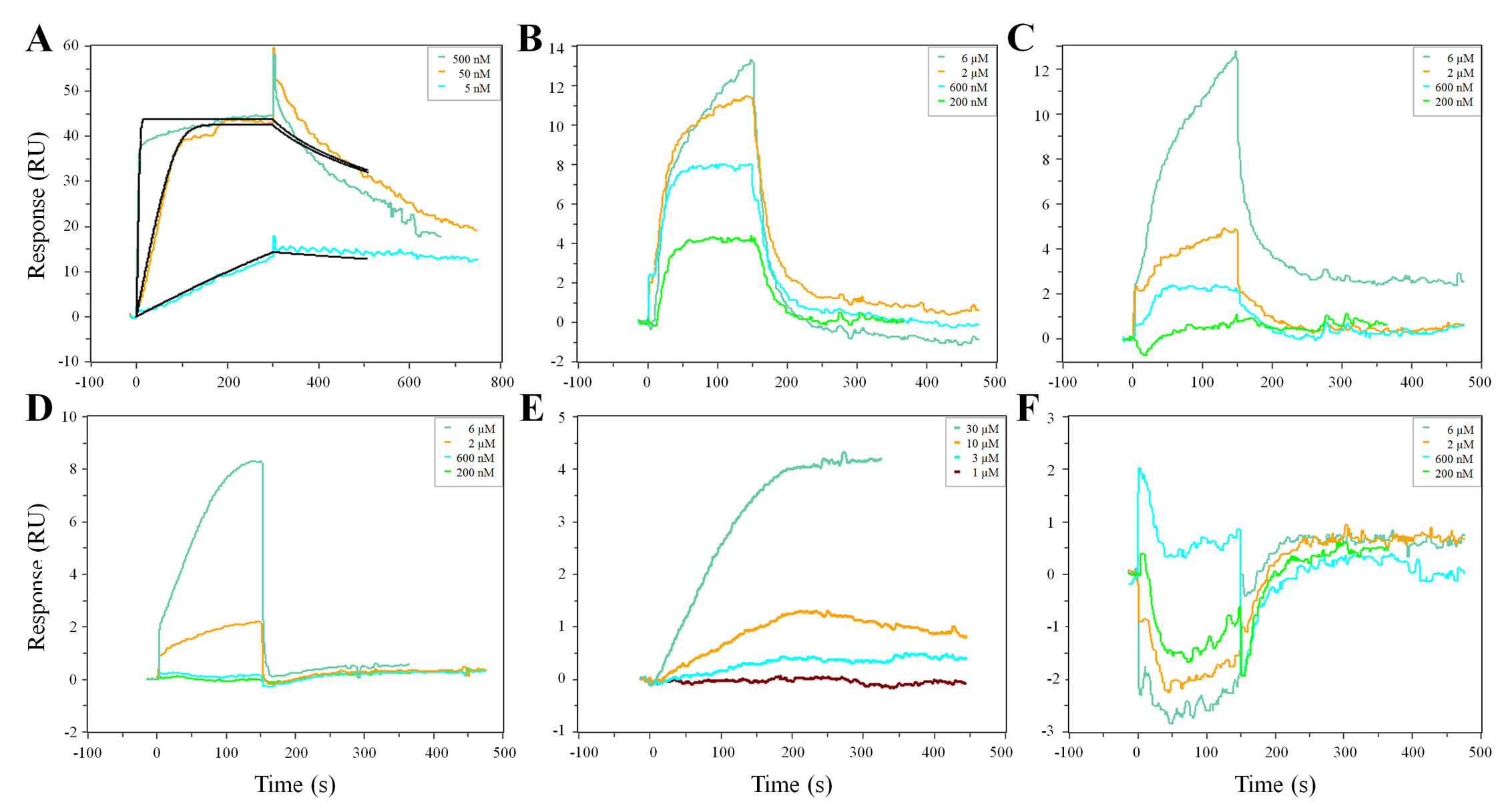

Supplement: S1 Fig — Time-dependent changes in resonance units (RU) were detected during the injection of (A) SB203580, (B) 3, (C) 9h, (D) 9l, (E) 9j and (F) 2 at various concentrations to a sensor surface carrying immobilized His6-p38α. Global 1:1-Langmuir binding fits are shown as black lines. (TIF) [file pone.0184627.s001.tif]

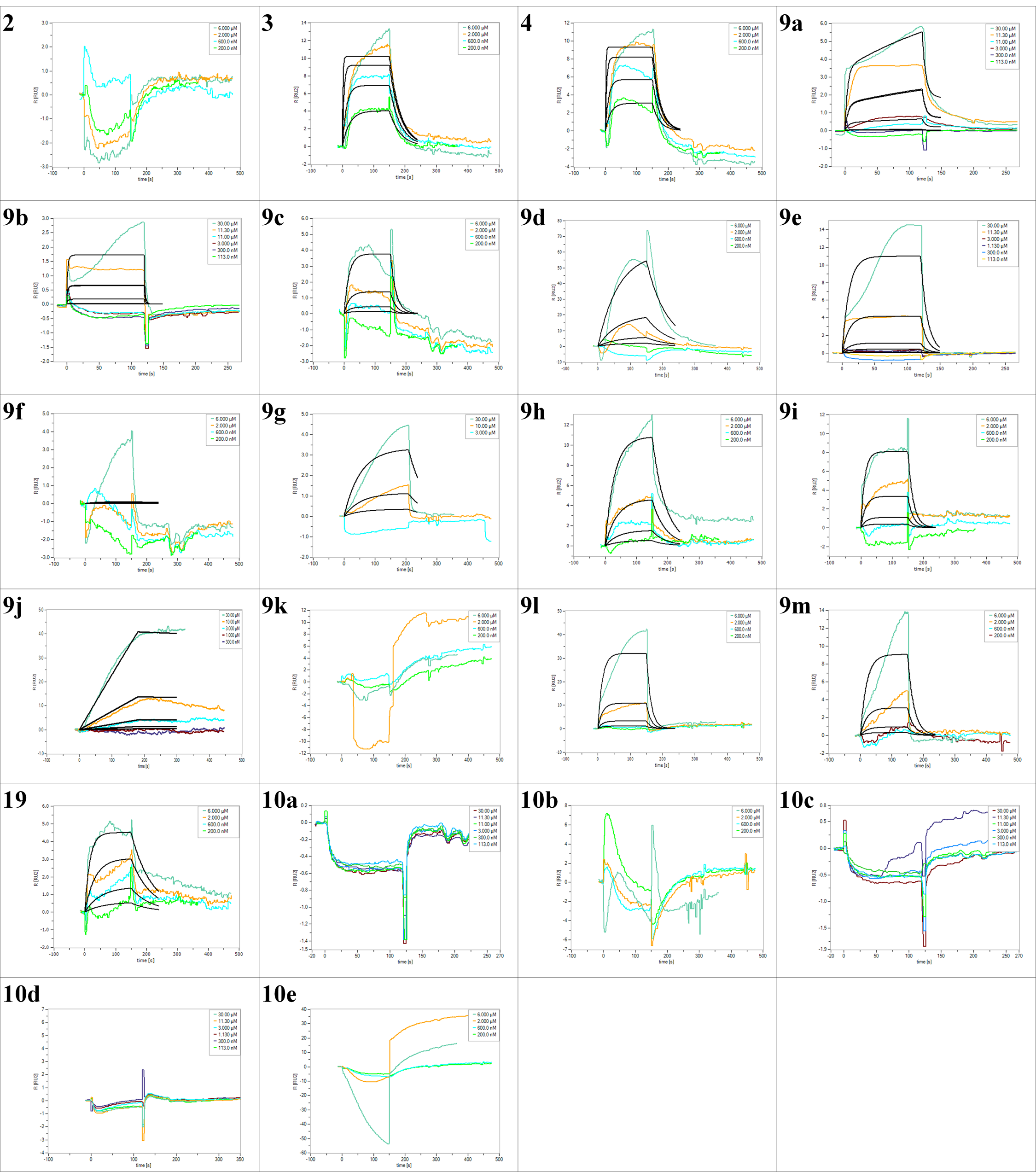

Supplement: S2 Fig — Time-dependent changes in resonance units (RU) were detected during the injection of LiPoLis in concentrations ranging from 1 nM—30 μM to a sensor surface carrying immobilized His6-p38α. Global 1:1-Langmuir and multi-phasic binding fits, respectively, are shown as black lines. LiPoLi nitro derivatives showed no response and are therefore not shown. (TIF) [file pone.0184627.s002.tif]

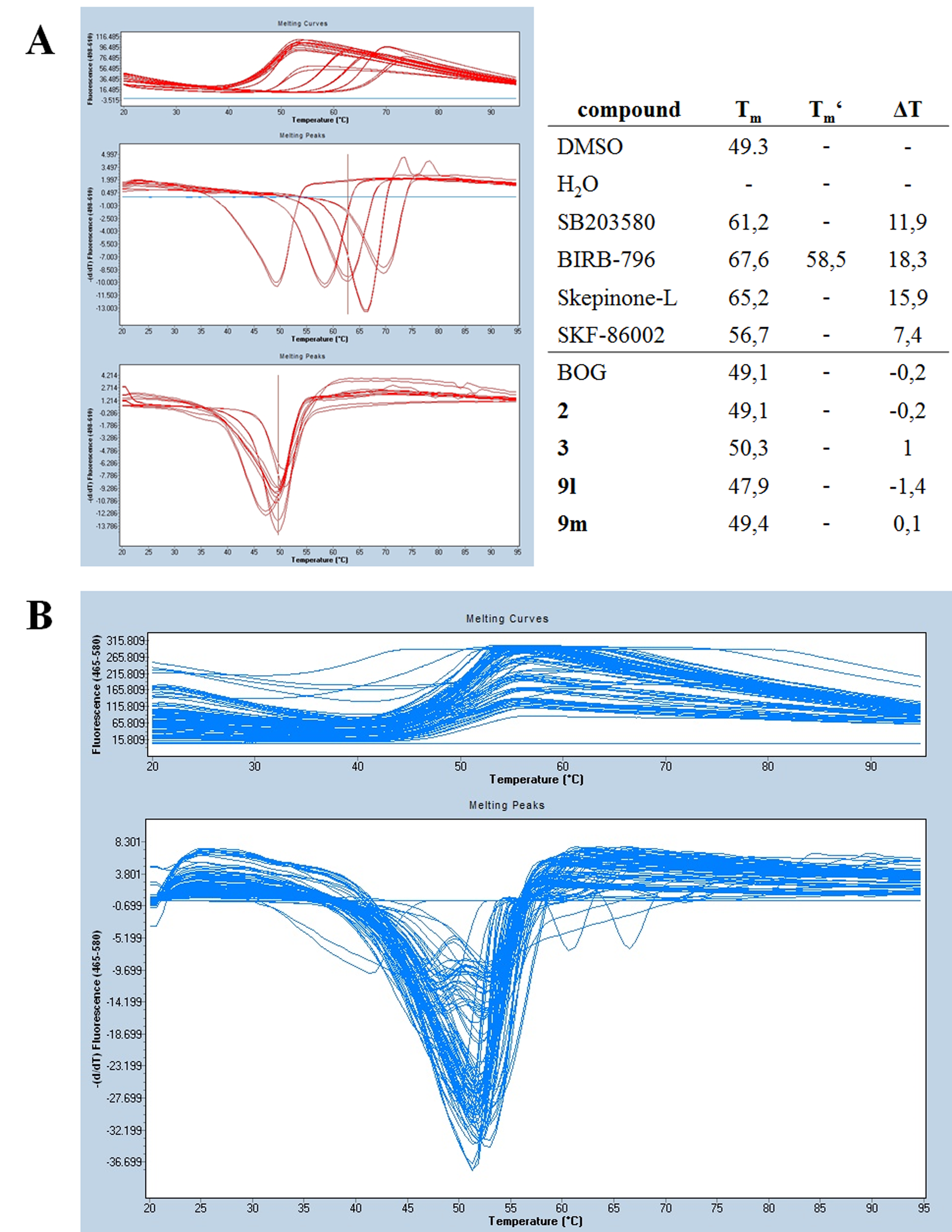

Supplement: S3 Fig — A) p38α MAPK melting curves and their first derivatives in presence of active site inhibitors and a selection of LiPoLis. Thermal shifts ΔT (°C) were calculated from substraction of melting point in presence of DMSO from measured melting points Tm (°C) in presence of compound. B) p38α MAPK melting curves and their first derivatives for all presented LiPoLis. (TIF) [file pone.0184627.s003.tif]

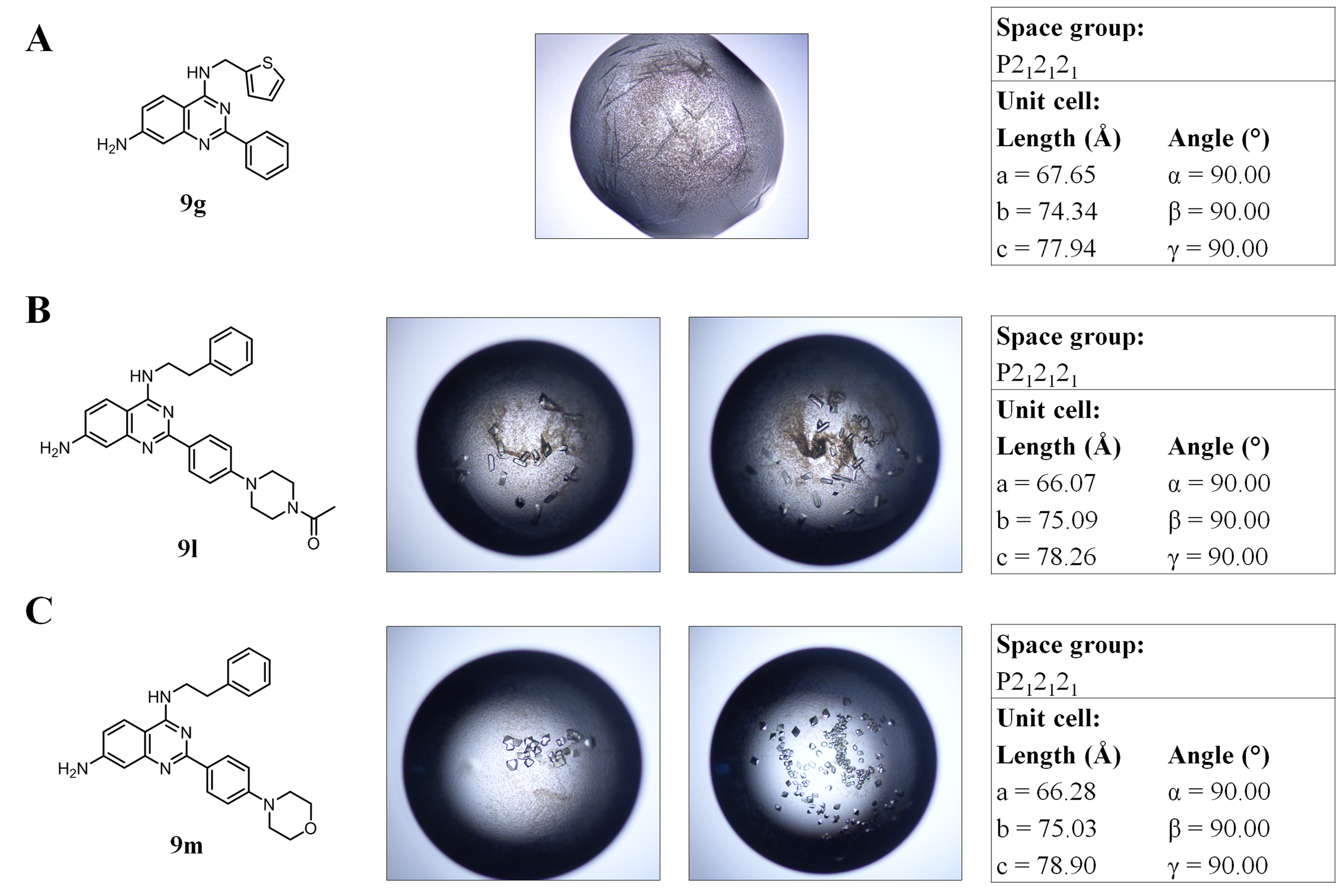

Supplement: S4 Fig — Crystals grown in presence of A) 9g (needles), B) 9l and C) 9m (cubic). Crystals were grown at 20°C using 100 mM MES pH 5.6–6.2, 20–30% PEG4000 and 50 mM BOG as reservoir solution. (TIF) [file pone.0184627.s004.tif]

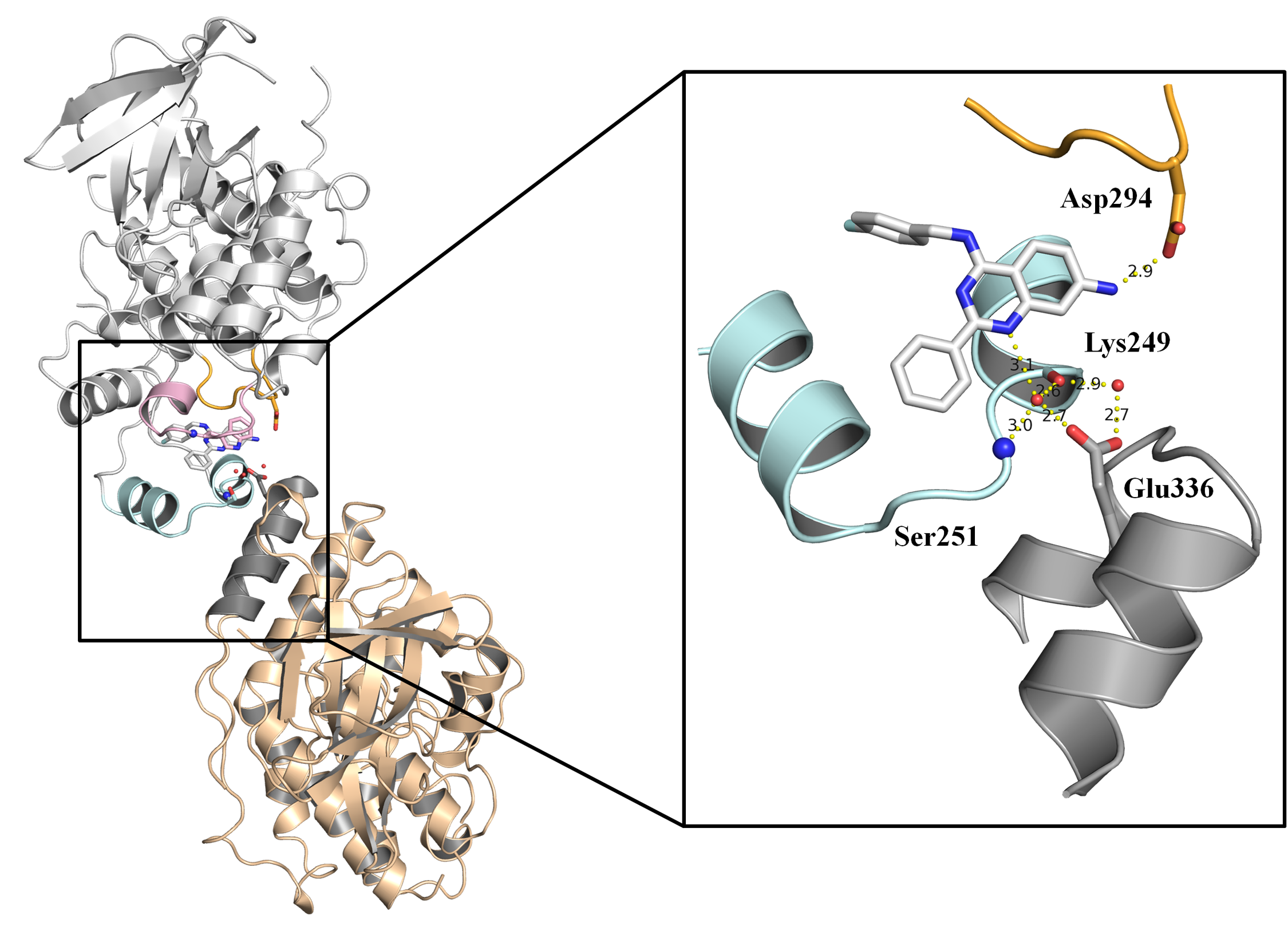

Supplement: S5 Fig — Exemplified for p38α in complex with 9c; symmetry mate is shown in grey, numbers represent H bond distances in Å (PDB: 5N63). (TIF) [file pone.0184627.s005.tif]

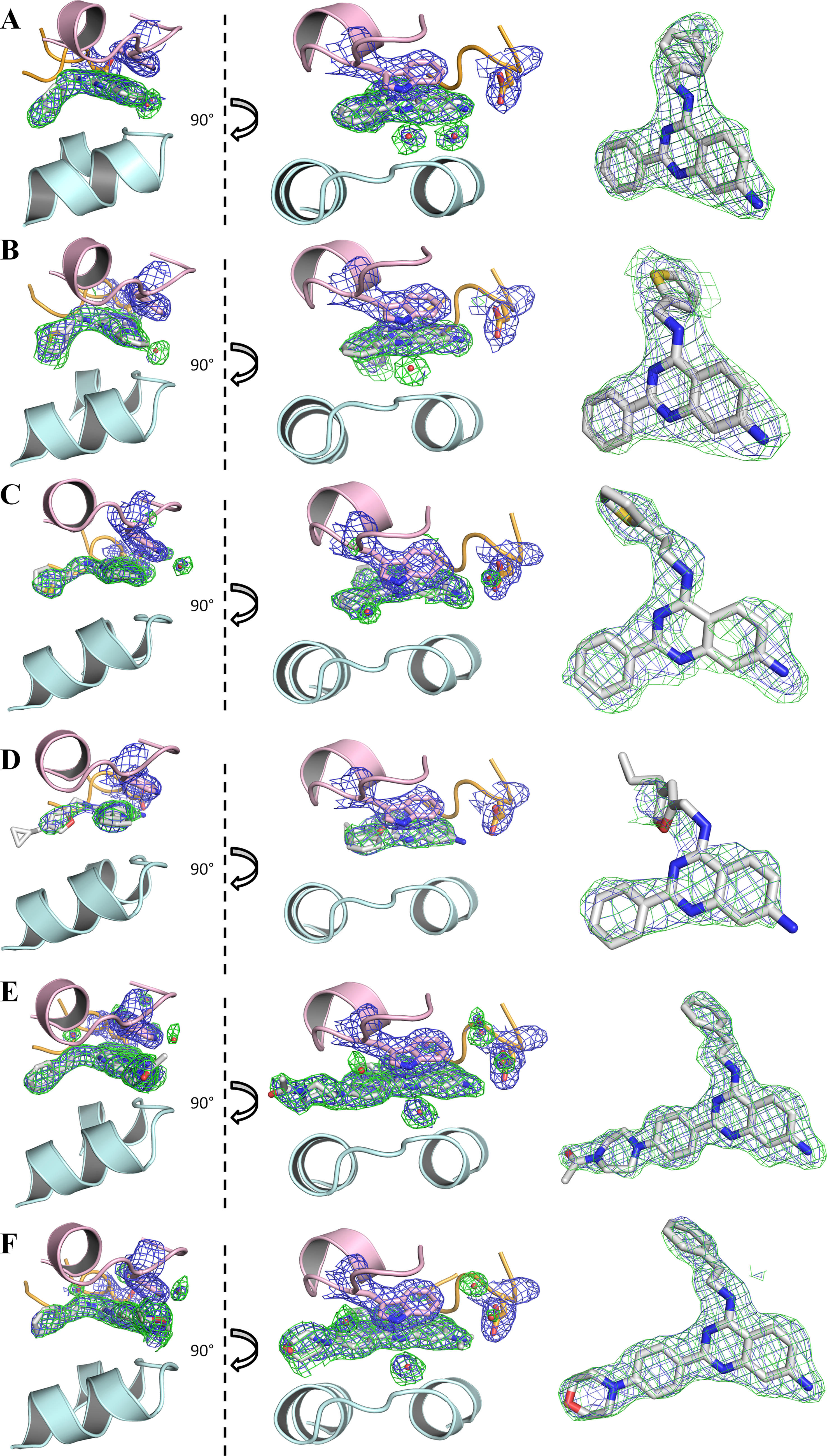

Supplement: S6 Fig — Performing a simulated annealing refinement, mFo-DFc omit maps (green, contoured at 2.5σ) were calculated for A) 9c, B) 9g, D) 9h, E) 9j, E) 9l and F) 9m, as well as for surrounding water molecules. 2Fo-Fc maps for the ligands, waters and key residues Trp197 and Asp294 were contoured at 1.0σ (blue). Maps indicate partial occupancy for 9h due to multiple molecules bound to the protein and conformational flexibility of the cyclopropyl moiety in 9j. (TIF) [file pone.0184627.s006.tif]

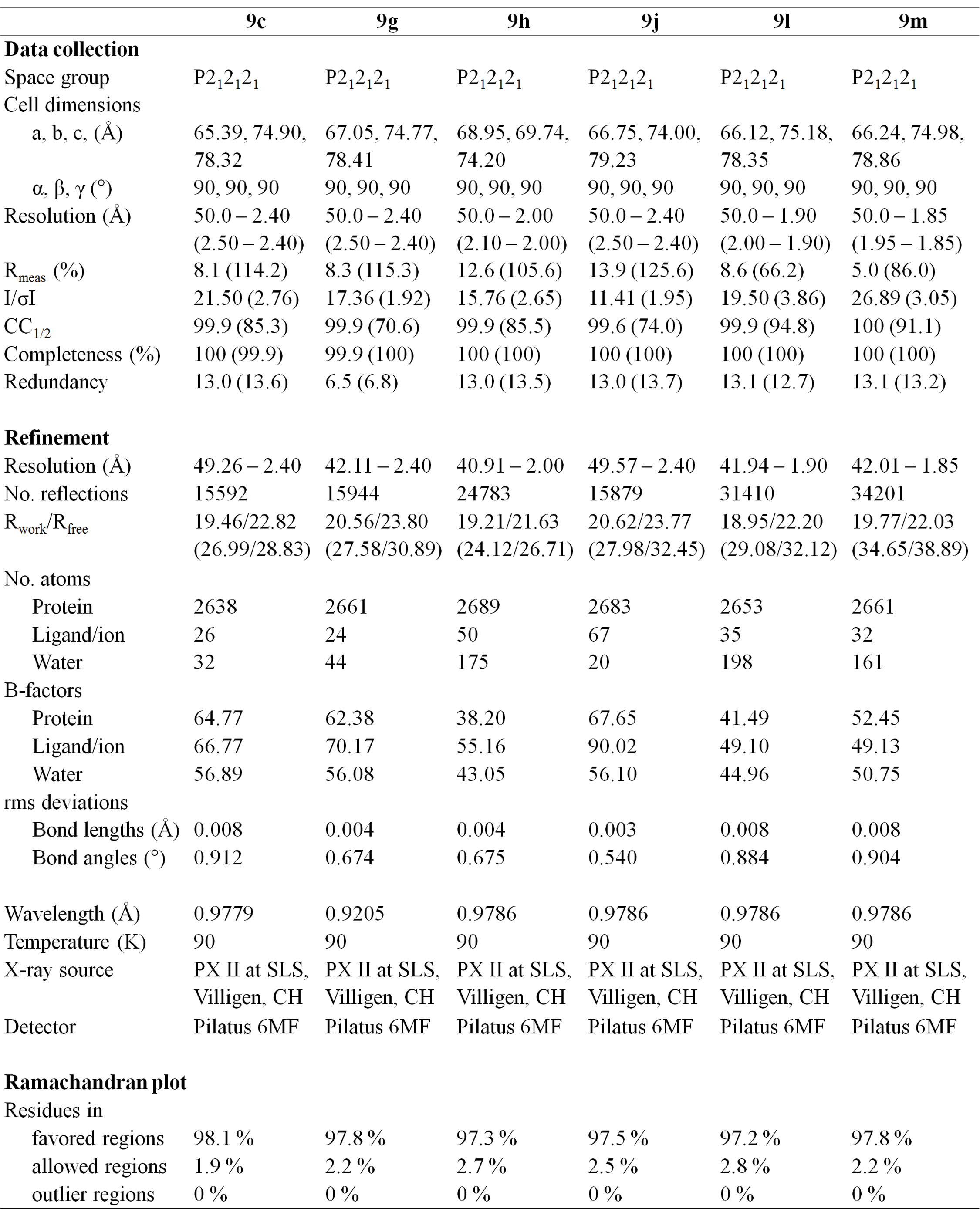

Supplement: S1 Table — Statistics for co-crystals with LiPoLis 9c, 9g, 9h, 9j, 9l and 9m (PDBs: 5N63, 5N64, 5N65, 5N66, 5N67 and 5N68). Values in parenthesis refer to the highest resolution shell. (TIF) [file pone.0184627.s007.tif]
