# Supplementary material for: Structure-based design, synthesis and crystallization of 2-arylquinazolines as lipid pocket ligands of p38α MAPK
Source: PLoS One. 2017 Sep 11;12(9):e0184627. doi: 10.1371/journal.pone.0184627 (PMC5593189; doi:10.1371/journal.pone.0184627)
Supplement: S2 File — (DOCX) [file pone.0184627.s009.docx]

**S1 Table. Overview of collected data regarding LiPoLi characterization.**

Overview of experimental results towards LiPoLi characterization. Following parameters are given in units shown in brackets: k_on_ (M · s)^‑1^, k_off_ (s^‑1^), *K*_D_ (M), *χ*^2^ (RU^2^). Those values were calculated according to 1:1‑Langmuir fits of the representative sensorgrams shown in S2 figure. In case of **9a** a heterogeneous fit model led to better fit quality and therefore, parameters representing multiple binding events indicated with 1 and 2, respectively, were correspondingly reported (S2 figure). As pointed out in the main text, all corresponding nitro derivatives did neither show response in SPR experiments nor led to crystal growth.

| **Compound** | **Structure** | **SPR*** | | | **Crystallization conditions** |
| --- | --- | --- | --- | --- | --- |
| **3** |  |  | k_on_  k_off_  *K*_D_  *χ*^2^ | = 8460  = 0.028  = 3.35 · 10^-5^  = 3.25 | morphology: needles  (+ BOG)  PDB: 4DLJ |
| **4** |  |  | k_on_  k_off_  *K*_D_  *χ*^2^ | = 9400  = 0.043  = 4.57 · 10^-5^  = 3.94 | morphology: needles  (+ BOG)  PDB; 4DLI |
| **9a** |  |  | k_on,1_  k_off,1_  *K*_D,1_  k_on,2_  k_off,2_  *K*_D,2_  *χ*^2^ | = 50.54  = 3.24 · 10^-3^  = 6.4 · 10^-5^  = 1869  = 0.203  = 1.09 · 10^-4^  = 0.80 | no crystals observed |
| **9b** |  |  | k_on_  k_off_  *K*_D_  *χ*^2^ | = 103.7  = 0.569  = 5.48 · 10^-3^  = 0.39 | no crystals observed |
| **9c** |  |  | k_on_  k_off_  *K*_D_  *χ*^2^ | = 1468  = 0.056  = 3.82 · 10^-5^  = 0.87 | morphology: needles  (w/o BOG)  PDB: 5N63 |
| **9d** |  |  | k_on_  k_off_  *K*_D_  *χ*^2^ | = 55.02  = 0.016  = 2.89 · 10^-4^  = 48.28 | no crystals observed |
| **9e** |  |  | k_on_  k_off_  *K*_D_  *χ*^2^ | = 61.07  = 0.096  = 1.57 · 10^-3^  = 1.79 | morphology: cubic  (+ BOG),  but no diffraction |
| **9f** |  | not determinable | | | no crystals observed |
| **9g** |  |  | k_on_  k_off_  *K*_D_  *χ*^2^ | = 17.58  = 0.019  = 1.07 · 10^-3^  = 0.63 | morphology: needles  (+ BOG)  PDB: 5N64 |
| **9h** |  |  | k_on_  k_off_  *K*_D_  *χ*^2^ | = 1911  = 0.023  = 1.21 · 10^-5^  = 0.62 | morphology: needles  (+ BOG)  PDB: 5N65 |
| **9i** |  |  | k_on_  k_off_  *K*_D_  *χ*^2^ | = 3976  = 0.059  = 1.49 · 10^-5^  = 1.60 | no crystals observed |
| **9j** |  |  | k_on_  k_off_  *K*_D_  *χ*^2^ | = 4.19  = 2.18 · 10^-4^  = 5.20 · 10^-5^  = 0.02 | morphology: needles  (+ BIRB-796)  PDB: 5N66 |
| **9k** |  | no binding | | | no crystals observed |
| **9l** |  |  | k_on_  k_off_  *K*_D_  *χ*^2^ | = 146.5  = 0.073  = 5.01 · 10^-4^  = 13.08 | morphology: cubic  (w/o BOG)  PDB: 5N67 |
| **9m** |  |  | k_on_  k_off_  *K*_D_  *χ*^2^ | = 209.7  = 0.050  = 2.38 · 10^-4^  = 2.40 | morphology: cubic  (w/o BOG)  PDB: 5N68 |
| **19** |  |  | k_on_  k_off_  *K*_D_  *χ*^2^ | = 7948  = 0.015  = 1.90 · 10^-6^  = 0.25 | no crystals observed |
| **10a** |  | no binding | | | no crystals observed |
| **10b** |  | no binding | | | no crystals observed |
| **10c** |  | no binding | | | no crystals observed |
| **10d** |  | no binding | | | no crystals observed |
| **10e** |  | no binding | | | no crystals observed |

* The given parameters were directly derived from the reported fit models. Of note, *χ*^2^ values represent the quality of the fit and therefore, the reliability of the kinetic data.
